# Neutrophil membrane engineered HucMSC sEVs alleviate cisplatin-induced AKI by enhancing cellular uptake and targeting

**DOI:** 10.1186/s12951-022-01574-8

**Published:** 2022-08-01

**Authors:** Peipei Wu, Yuting Tang, Can Jin, Min Wang, Linli Li, Zhong Liu, Hui Shi, Zixuan Sun, Xiaomei Hou, Wenya Chen, Wenrong Xu, Hui Qian

**Affiliations:** 1 Zhenjiang Key Laboratory of High Technology Research on Exosomes Foundation and Transformation Application, 301 Xuefu Road, Zhenjiang, 212013 Jiangsu People’s Republic of China; 2 Jiangsu Key Laboratory of Medical Science and Laboratory Medicine, School of Medicine, Jiangsu University, 301 Xuefu Road, Zhenjiang, 212013 Jiangsu People’s Republic of China; 3 Department of Orthopedics, School of Medicine, Shanghai General Hospital, Shanghai Jiao Tong University, Shanghai, 200080 China; 4 NHC Key Laboratory of Medical Embryogenesis and Developmental Molecular Biology & Shanghai Key Laboratory of Embryo and Reproduction Engineering, Shanghai, 200040 China

**Keywords:** Mesenchymal stem cells, Engineered sEVs, Neutrophil membrane, Antioxidant, Acute kidney injury

## Abstract

Human umbilical cord mesenchymal stem cells-derived small extracellular vesicles (hucMSC-sEVs) have been demonstrated as a therapeutic agent to prevent and treat cisplatin-induced acute kidney injury (AKI). However, hucMSC-sEVs still face many problems and challenges in the repair and treatment of tissue injury, including short circulation time, insufficient targeting, and low therapeutic efficacy. Therefore, we constructed engineered hybrid vesicles fused with nanovesicles derived from human neutrophil membranes and hucMSC-sEVs, named neutrophil membrane engineered hucMSC-sEVs (NEX). NEX significantly enhanced the targeting of hucMSC-sEVs to injured kidney tissues, improved the impaired renal function via reducing pro-inflammatory cytokines expression, promoted the proliferation of renal tissue cells, and inhibited renal cell apoptosis in vivo. In addition, NEX enhanced hucMSC-sEVs uptake by NRK52E cells, but inhibited its uptake by RAW264.7 cells. Moreover, administration of NEX reduced cellular oxidative stress and promoted proliferation of NRK52E cells treated with cisplatin in vitro. In summary, our findings indicate that this design of a universal approach enhances the targeting and therapeutic efficacy of hucMSC-sEVs in kidney tissue regeneration, and provides new evidence promoting its clinical application.

**Supplementary Information:**

The online version contains supplementary material available at 10.1186/s12951-022-01574-8.

## Background

Acute kidney injury (AKI) is a clinical syndrome caused by rapid decline of renal function due to a variety of pathogenic factors [1]. AKI is one of the most common acute complications with high morbidity and mortality. Its pathogenesis includes inflammatory imbalance, abnormal hemodynamics, excessive reactive oxygen species (ROS) production [2]. Currently, there are no effective drugs to prevent or treat AKI. Thus, there is an urgent need to find an effective approach for prevention and treatment. In recent years, mesenchymal stem cells (MSCs) based on stem cell transplantation therapy have aroused extensive interest in the prevention and treatment of various diseases, such as ischemic myocardial infarction, acute and chronic liver and kidney injury, liver and kidney fibrosis, skin wound healing, bone and cartilage regeneration, spinal cord injury, psoriasis, and systemic lupus erythematosus [3, 4]. MSCs play a role in complex and refractory diseases through directly differentiating into damaged cells or paracrine, as well as immune regulation. However, for in vivo infusion of MSCs, long-term safety, storage, and transportation difficulties limit clinical application. Therefore, there is an urgent need to find new biomaterials and stem cell derivatives to replace MSCs in tissue regeneration and repair.

Exosomes, as a type of small extracellular vesicle (sEV), contain a variety of bioactive molecules such as proteins, nucleic acids, lipids, and metabolites that not only mediate cell-to-cell signal communication but also participate in the regulation of various physiological and pathological processes, including immune response, neuronal communication, antigen presentation, organ development, and regeneration. sEVs derived from normal tissue epithelial cells, stem cells, immune cells, prokaryotic cells, and other sources have been found to be involved in the regulation of various pathological processes. sEVs, as an important paracrine component of MSCs, have been shown to play beneficial roles in various diseases, such as cardiac repair [5], liver injury [6], kidney injury [7], wound healing [8], autoimmune and neurodegenerative disorders diseases [9], spinal cord injury [10], diabetes [11], and other diseases [12]. Our previous studies have proved that 14-3-3ζ carried by human umbilical cord mesenchymal stem cells derived small extracellular vesicles (hucMSC-sEVs) have a significant therapeutic effect on tissue regeneration, including cisplatin-induced AKI [13–16]. Moreover, hucMSC-sEVs deliver miR-125-5P [17] and miR-146a-5p to repair ischemic-induced AKI [18]. Other studies have also shown that some miRNA molecules, such as miR-22-3p [19], miR-30C-5p [20], and miR-150-5p [21], are significantly down-regulated in septic-induced AKI, and their overexpression plays a significant role in renal protection. However, the short circulation time, poor biochemical stability, insufficient targeting, and low therapeutic efficacy of sEVs greatly limit their clinical application in the process of tissue regeneration and repair [22].

In this study, we used the fusion technology of neutrophil membranes and hucMSC-sEVs to obtain engineered NEX and explore its role and mechanism in the treatment of an acute kidney injury rat model. We found that the engineered NEX significantly promoted its targeted enrichment in damaged renal tissue and improved acute kidney injury. Its mechanism is mainly manifested in inhibiting inflammatory response, promoting cell proliferation, reducing apoptosis and oxidative stress, and preventing the clearing of macrophages.

## Materials and methods

### Ethics

All studies were approved by the Medical Ethics Committee of Jiangsu University (IRB approval protocol number: 2020161).

### Cell culture

Primary hucMSCs were isolated as previously described [23]. Fresh umbilical cord tissues were obtained from the Fourth Affiliated Hospital of Jiangsu University after the informed consent of the puerpera. Briefly, the umbilical cord tissues were washed and cut into 2-cm pieces and then placed in phosphate buffer solution (PBS) containing penicillin–streptomycin for approximately 15 min. Subsequently, the arteries and veins of the umbilical cord tissue were removed and processed into tissue blocks of approximately 3 mm^3^. The small tissue blocks were pasted on the bottom of a petri dish and then placed upside-down in a cell culture incubator for approximately 30–60 min. Subsequently, the umbilical cord tissues were maintained in α-MEM medium (Invitrogen, USA) containing 20% FBS (Bovogen, Australia) containing penicillin–streptomycin (Leagene, China). The medium was changed every 3 days for approximately 10 days. The passaged cells were maintained with α-MEM containing 10% FBS and penicillin–streptomycin, and the P3 hucMSCs were used for subsequent experimental studies. Rat renal tubular epithelial cell NRK52E and mouse mononuclear macrophage RAW 264.7 cells were purchased from American Type Culture Collection. The NRK52E cells were cultured in high-glucose DMEM (Bioind, Israel) with 15% FBS, and the RAW 264.7 cells were cultured in RPMI 1640 medium (Invitrogen, USA) with 10% FBS. All cells were maintained in a humidified incubator with 5% CO_2_ at 37 °C.

### Identification of hucMSCs

For the identification of multi-directional differentiation potential, the P3 hucMSCs were inoculated into 6-well plates at a density of 2 × 10^4^ cells/cm^2^. According to the manufacturer’s instructions to perform adipogenic differentiation (Cyagen Biosciences, HUXUC-90031, USA). Briefly, when the degree of cell fusion reached 100%, 2 mL of adipogenic differentiation medium A was added into each well for 3 days, and then 2 mL of adipogenic differentiation medium B was replaced for 1 day. The above steps were repeated until the cells were cultured alternately for approximately 12–20 days. Oil red O staining was applied to evaluate the adipogenic differentiation of the hucMSCs. According to the manufacturer's instructions to perform osteogenic differentiation (Cyagen Biosciences, HUXUC-90021, USA). Briefly, when the degree of cell fusion reached 60–70%, 2 mL of osteogenic differentiation medium was added into each well, which was replaced with fresh complete medium every 3 days. The above steps were repeated until the cells were cultured for approximately 14 days. Alizarin Red S staining was performed to identify the osteogenic differentiation of the hucMSCs. According to the manufacturer’s instructions to detect the surface markers of hucMSCs (Cyagen Biosciences, HUXMX-09011, USA). The P3 hucMSCs suspension (3 × 10^6^ cells/mL) was randomly divided into 1.5-mL sterile EP tubes. The primary antibodies, including isotype control, anti-human CD11b, anti-human CD14, anti-human CD45, anti-human CD29, anti-human CD73, and anti-human CD105, were added to each tube separately and incubated in the dark for 30 min. After the cells were washed twice by flow cytometry buffer, PE-labeled fluorescence secondary antibody was added to each tube, and incubated in the dark for 30 min. Ultimately, flow cytometry was performed to detect the surface markers of the hucMSCs.

### Isolation and identification of hucMSC-sEVs

The hucMSC-sEVs were isolated and purified as previously described [24, 25]. The conditioned medium of P3-6 hucMSCs with good growth condition was collected. Cell supernatants were centrifuged to remove cell debris and organelles. Finally, the exosome pellets were resuspended in PBS and then passed through a 0.22-μm filter (Millipore, USA) and stored at − 80 °C. The protein content of the hucMSC-sEVs was determined by using a BCA protein assay kit (Vazyme, Nanjing, China). The morphology of the hucMSC-sEVs was observed using transmission electron microscopy (TEM; H-7800, Hitachi, Japan). The particle size, concentration, and zeta potential of the hucMSC-sEVs were analyzed by Nanoparticle tracking analyzer (NTA) (Germany, Particle Metrix, 220-Twin). The positive markers of hucMSC-sEVs, such as CD9, CD63, CD81, TSG101, Alix, and HSP70, as well as the negative control Calnexin, were determined by western blotting.

### Isolation and identification of human peripheral blood neutrophils

Fresh anticoagulant blood samples from healthy volunteers were collected, and 5 mL of fresh whole blood was slowly added along the tube wall into a 15-mL sterile centrifuge tube with 5 mL of PolymorphPrep separation solution at the bottom under sterile conditions. After centrifugation at 600*g* for 30 min at 23 ℃, a pipette was used to carefully remove the intermediate white film layer into serum-free 1640 medium for washing. After centrifugation at 800*g* for 5 min at 23 ℃, the supernatant was discarded, and freshly configured red blood cell lysis buffer was added to resuspend the cell precipitate. Then, the cells were gently blown and mixed, and they stood at room temperature for 5–10 min. The cells were terminated with serum-free 1640 medium. After centrifugation at 800*g* for 5 min at 23 ℃, the neutrophils precipitation was harvested, and precooled PBS was washed three times and centrifuged at 800*g* for 5 min at 4 ℃. Subsequently, the neutrophils cell precipitation was obtained. Phase contrast microscopy was applied to observe the morphological characteristics of the neutrophils. Wright–Giemsa staining was performed to detect the nuclear and cytoplasmic characteristics of the neutrophils. The surface specific antigen molecules CD11b and CD33 of the neutrophils were identified by laser scanning confocal microscopy and flow cytometry. The integrity of the plasma membrane of DIO and DIL stained neutrophils was determined by laser scanning confocal microscopy.

### Cell membrane derivation

The plasma membranes of the neutrophils were extracted as previously described with added modifications [25–27]. Briefly, frozen cells were thawed and washed with precooled 1 × PBS three times before centrifuging at 800*g* for 10 min. The cells were suspended in isolation buffer 1 containing 225 mM mannitol, 75 mM sucrose, 0.5% (wt/vol) BSA, 0.5 mM EGTA, and 30 mM Tris–HCl at pH 7.4 (all reagents from Sigma), as well as a protease and phosphatase inhibitor cocktail (CST, USA, 5870S). The cells were broken using a tissue dissociator, and then the homogenized solution was centrifuged at 800*g* for 10 min at 4 °C. Subsequently, the pellet was discarded, and the supernatant was centrifuged at 10,000*g* for 30 min at 4 °C. Following the centrifugation, the pellet was discarded, and the supernatant was centrifuged at 100,000*g* for 2 h at 4 °C. Finally, the membranes were collected as a pellet and washed twice with 0.2 mM EDTA in water. The membrane protein content of the neutrophils was determined by using a BCA protein assay kit. Ultimately, the membranes were stored at − 80 °C for subsequent studies.

### Preparation and identification of Neu-NVs and NEX

Nanovesicles derived from human neutrophil membrane (Neu-NVs) were synthesized using sonication combined with extrusion. Briefly, the plasma membrane solution of neutrophils was ultrasonicated in an ice bath for 2 min. Subsequently, in order to prepare the Neu-NVs, the membrane protein components were successively extruded through 400 nm, 200 nm, and 100 nm polycarbonate membranes of liposome extruder (Germany, Merck, Avanti) 33 times. In order to prepare a NEX hybrid of Neu-NVs and hucMSC-sEVs, Neu-NVs and hucMSC-sEVs were mixed at a ratio of 1:1, and the mixture was then sonicated in an ice bath for 2 min. Furthermore, the mixture was then successively extruded through 400 nm, 200 nm, and 100 nm polycarbonate membranes of liposome extruder for a total of 33 times to form NEX. TEM (Transmission electron microscope), AFM (Atomic force microscope), and NTA were performed to detect the size, morphology, appearance, height, particle size, concentration, and zeta potential of the Neu-NVs and NEX. Western blotting was employed to determine the specific protein markers of the neutrophils. Coomassie blue staining was used to evaluate the protein components of the hucMSC-sEVs and the neutrophil membranes.

### Membrane fusion verification

The fusion of Neu-NVs and hucMSC-sEVs was first investigated by a FRET(Fluorescence resonance energy transfer) method [28, 29]. Briefly, the hucMSC-sEVs were simultaneously labeled with DIO (excitation/emission = 480/510 nm) and DIL (excitation/emission = 549/565 nm) membrane dyes. The DIO-DIL-labeled hucMSC-sEVs and Neu-NVs were mixed by sonication combined extrusion to facilitate membrane fusion at a ratio of 1:1. The fluorescence spectrum of each sample was recorded between 490 and 650 nm using a Cytation 5 automatic microplate reader (BioTek, USA) with an excitation wavelength of 470 nm. For fluorescence colocalization experiments, DIO-labeled Neu-NVs and DIL-labeled hucMSC-sEVs were mixed at a ratio of 1:1 by simply stirring or the ultrasound combined extrusion method to facilitate membrane fusion. The fusion of Neu-NVs and hucMSC-sEVs was detected by super-resolution microscopy.

### Mouse model of AKI and therapeutic experiments

Male ICR mice (8 to 10 weeks old, weighing 20 g ± 2 g) were purchased from the Laboratory Animal Center of Jiangsu University (Zhenjiang, China) and randomly divided into five groups (n = 6). All animal experiments were compliant with standard guidelines for the care and use of laboratory animals and were approved by the Institutional Animal Care and Use Committees of Jiangsu University. A mouse model of cisplatin-induced AKI was established as described previously. After 10 mg/kg cisplatin (Sigma-Aldrich, USA, P4394) intraperitoneal injection for 72 h, Neu-NVs, hucMSC-sEVs, and NEX with the same particle number were administered to the AKI mice by tail vein for 2 consecutive days. AKI mice injected with PBS were used as a positive control. A normal group without any treatment served as a negative control. All animals were sacrificed at day 6 after cisplatin injection. Serum and kidney tissues were removed for further analysis of the renal function, histological changes, and tubular apoptosis.

### Small animal in vivo imaging

One milliliter volumes of Neu-NVs, hucMSC-sEVs, and NEX were incubated with 5 μL of the membrane dye DIL (Thermo, USA, D3911) for 30 min at 37 °C. The labeled three types of vesicle suspension were transferred to a 100-kDa MWCO ultrafiltration centrifugal tube (Millipore, USA). In order to remove the unconjugated DIL, the samples were washed with PBS 3 times and centrifuged at 1500×*g* for 30 min. DIL-labeled Neu-NVs, hucMSC-sEVs, and NEX with the same particle number were administered to the AKI mice by tail vein, and small animals in vivo imaging (PerkinElmer, USA) was used to detect the major tissue organs distribution of the three types of transplanted vesicles at 24 h.

### HE, IHC, and TUNEL staining

Bilateral renal tissues were obtained from the sacrificed mouse. The samples were fixed in 4% paraformaldehyde and gradually dehydrated, embedded in paraffin, and then cut into 4-μm sections. The slides were dewaxed to water and prepared via Hematoxylin and Eosin (HE) staining in accordance with standard protocols. Immunohistochemistry (IHC) staining was performed in accordance with the manufacturer’s instructions (Boster, USA, SA1020). PCNA primary antibody (CST, USA, 4711S) was diluted with 5% BSA at the ratio of 1:50, and then was incubated with sections for IHC. The stained slides were sealed and dried for observation. The apoptotic renal cells in tissue slides were measured by using terminal deoxynucleotidyl transferase-mediated dUTP nick end-labeling (TUNEL) staining according to the manufacturer’s protocol (Vazyme, USA, A113-02).

### Quantification of serum cytokines

AKI mouse serum samples were collected on day 6, and the concentrations of IFN-α, IFN-γ, TNF-α, IL-1β, IL-2, IL-4, IL-5, IL-6, IL-8, IL-17, IL-10, and IL-12P70 were quantified with a multifunctional flow cytometer. Specifically, the whole blood of the AKI mice was collected and allowed to clot at room temperature for 30 min. The samples were then centrifuged at 2000*g* for 10 min to collect serum from the supernatant. The serum samples were immediately frozen at − 80 °C until they were analyzed via multiparameter flow cytometry within 3 days of collection.

### Quantitative real-time polymerase chain reaction (qRT-PCR)

Trizol reagent (Invitrogen, MA, USA) was used for total RNA extraction from cells, followed by cDNA synthesis with a commercial reverse transcription kit (Vazyme, Nanjing, China) according to the manufacturer’s protocol. On a QuantStudio™ 3 Real-Time PCR detection system (ABI, USA), qRT-PCR was run with SYBR Green PCR kit (CWBIO). The changes in the mRNA levels of the samples were evaluated using the 2^−ΔΔ^ Ct method and were relative to the β-actin levels. The PCR primer sequences of the genes are listed in Additional file 1: Table S1.

### Cell proliferation assay

The NRK52E cells (5 × 10^3^ cells/well) were seeded in 96-well plates. After the cells adhered for 12–24 h, different concentrations of cisplatin were added to each well for treatment for 12 h and 24 h. The Cell Counting Kit-8 (CCK-8) assay was performed to evaluate cell proliferation activity according to the manufacturer’s procedures. One hundred µL of medium containing 10% CCK-8 reagent (Vazyme, Nanjing, China) was added to each well and incubated in the dark for 2 h at 37 °C. The absorbance values of each well at 450 nm were measured by the Cytation 5 automatic microplate reader (BioTek, USA). The NRK52E cells were seeded in 6-well plates at a density of 2 × 10^4^ cells/cm^2^. When the cell density reached 80–90%, Neu-NVs, hucMSC-sEVs, and NEX with the same particle number were added to the cells and treated with cisplatin at a concentration of 10 ng/mL for 12 h. The cell proliferation activity was evaluated according to the manufacturer’s protocol by using an EDU (5-Ethynyl-2′-deoxyuridine) staining proliferation detection kit (Beyotime, Shanghai, China). After staining, the cells were immediately detected for green fluorescence intensity at 519 nm using the Cytation 5 automatic microplate reader (BioTek, USA).

### Western blotting

Cells and exosomes were lysed in radio-immunoprecipitation assay (RIPA) buffer containing proteinase inhibitors (Pierce). The total protein concentration was detected using a BCA protein assay kit. An equal amount of extracted protein was separated by 12% SDS–polyacrylamide gel electrophoresis (SDS-PAGE) and transferred to PVDF membranes (Millipore, MA, USA) followed by blocking with 5% nonfat milk for 1 h. Afterward, the membranes were incubated with primary antibodies at 4 °C overnight. The membranes were washed with 1xTBST and then incubated with an HRP-conjugated goat anti-rabbit/mouse IgG secondary antibody (USA, Invitrogen, 31460/31430) for 1 h at room temperature. The primary antibodies used in this study were: CD9 (Proteintech, USA, 60232-1-Ig), CD63 (Abcam, USA, ab59479), CD81 (Proteintech, USA, 18250-1-AP), HSP70 (CST, USA 2679S), Alix (CST, USA, 2171S), TSG101 (Abcam, ab30871), Calnexin (CST, USA, 4872S), CCL2 (Abclonal, USA, A7277), CXCR4 (Abcam, USA, ab124824), Fas (Absin, China, abs115046), ICAM-1 (Abcam, ab179707), integrin αV (CST, 4711S), integrin β3 (CST, 4702S), and β-actin (Bioworld, USA, AP0060).

### Cellular uptake experiment

One-milliliter volumes of hucMSC-sEVs and NEX were incubated with 5 μL of the membrane dye DIO (Thermo, V22886) for 30 min at 37 °C. The stained hucMSC-Ex and NEX were then transferred to 100-kDa MWCO ultrafiltration centrifugal tubes (Millipore, USA). In order to remove the unconjugated DIO, the samples were washed three times with PBS and centrifuged at 1500*g* for 20 min. DIO-labeled Neu-NVs, hucMSC-sEVs, and NEX with the same particle number were administered to the NRK52E cells and RAW264.7 cells, and small animals in vivo imaging (PerkinElmer, USA) was used to detect the major tissue organ distribution of the three types of transplanted vesicles at 24 h. Laser scanning confocal microscopy and flow cytometry were applied to identify the internalization of the DIO-labeled hucMSC-sEVs and NEX by the NRK52E and RAW264.7 cells at different time points.

### Cell ROS detection

The NRK52E cells were seeded in six-well plates at a density of 2 × 10^4^ cells/cm^2^. When the cell density reached 80–90%, Neu-NVs, hucMSC-sEVs and NEX with the same particle number were added to cells and treated with cisplatin at a concentration of 10 ng/mL for 12 h. The intracellular ROS expression levels in each group were evaluated according to the manufacturer’s procedures. One milliliter of serum-free medium containing 10 μmoL DCFH-DA working solution (Beyotime) was added to each well and incubated in the dark for 20 min at 37 °C. The cells were washed in serum-free medium three times and were immediately detected for green fluorescence intensity at 525 nm using the Cytation 5 automatic microplate reader (BioTek, USA).

### Statistical analysis

All data are presented as mean ± standard deviation (SD). Statistical analyses were performed with GraphPad Prism software (Version 5.01). The statistically significant differences between any two groups were determined using the two-tailed unpaired Student’s *t*-test. Comparisons between more than two groups were analyzed using one-way analysis of variance (ANOVA). *P* value < 0.05 was considered statistically significant. *P < 0.05, **P < 0.01, and ***P < 0.001.

## Results

### Characterization of HucMSCs and hucMSC-sEVs

**Fig. 1:**
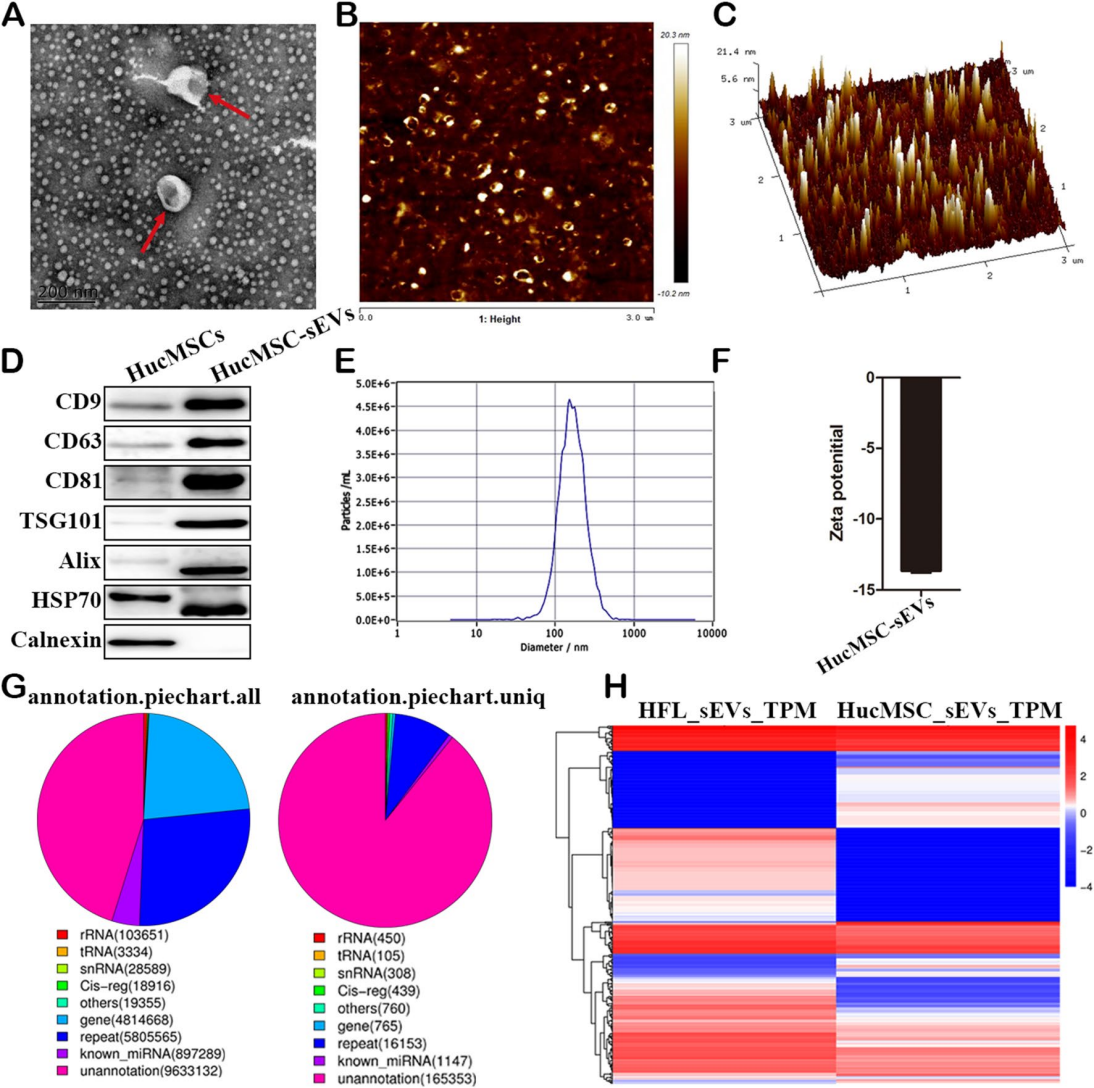
Characterization of hucMSC-sEVs. **a** The size and morphology were measured by TEM (scale: **200** nm). **b**, **c** The appearance and height of the hucMSC-sEVs were determined by AFM. **d** The expression of positive and negative characteristic markers of the hucMSC-sEVs were detected by western blotting. **e**, **f** Particle size, concentration, and zeta potential of hucMSC-sEVs determined by NTA. **g**, **h** MiRNA sequencing was performed to evaluate small RNA molecules enriched in the hucMSC-sEVs

Primary hucMSCs were isolated as previously described [23]. The morphology of the primary hucMSCs was a long spindle shape, and the growth of hucMSCs adhered to the wall in a vortex shape (Additional file 1: Fig. S1a). The results of adipogenic-induced differentiation showed that many orange-red lipid droplets of different sizes, rounds, or ovals appeared in the hucMSCs (Additional file 1: Fig. S1b). The results of osteogenic-induced differentiation showed that the typical red calcium nodule structure appeared in the hucMSCs (Additional file 1: Fig. S1b). The hucMSCs were positive for CD29, CD73, and CD105 and negative for CD11b, CD14, and CD45 (Additional file 1: Fig. S1c). The hucMSC-sEVs were extracted as previously described [18, 19]. The TEM and AFM results showed that the hucMSC-sEVs had a typical cup-like structure (Fig. 1a–c), and their heights were varied but mainly concentrated around 10 nm (Fig. 1c). Moreover, the hucMSC-sEVs expressed characteristic proteins such as CD9, CD63, CD81, TSG101, Alix, and HSP70, while they negatively expressed calnexin (Fig. 1d). The particle size of the hucMSC-sEVs was mainly distributed in the range of 30–200 nm, with the peak concentrated at 154 nm (Fig. 1e), and the hucMSC-sEVs presented a negative zeta potential (Fig. 1f). Our and other studies have shown that 14-3-3ζ protein, miR-125-5p, and miR-146a-5p enriched in hucMSC-sEVs can be used to alleviate AKI caused by different pathological factors (Additional file 1: Fig. S2b). Again, miR-22-3p, miR-30c-5p, and miR-150-5p have been reported to effectively repair sepsis-induced AKI.

### Preparation and identification of Neu-NVs

**Fig. 2:**
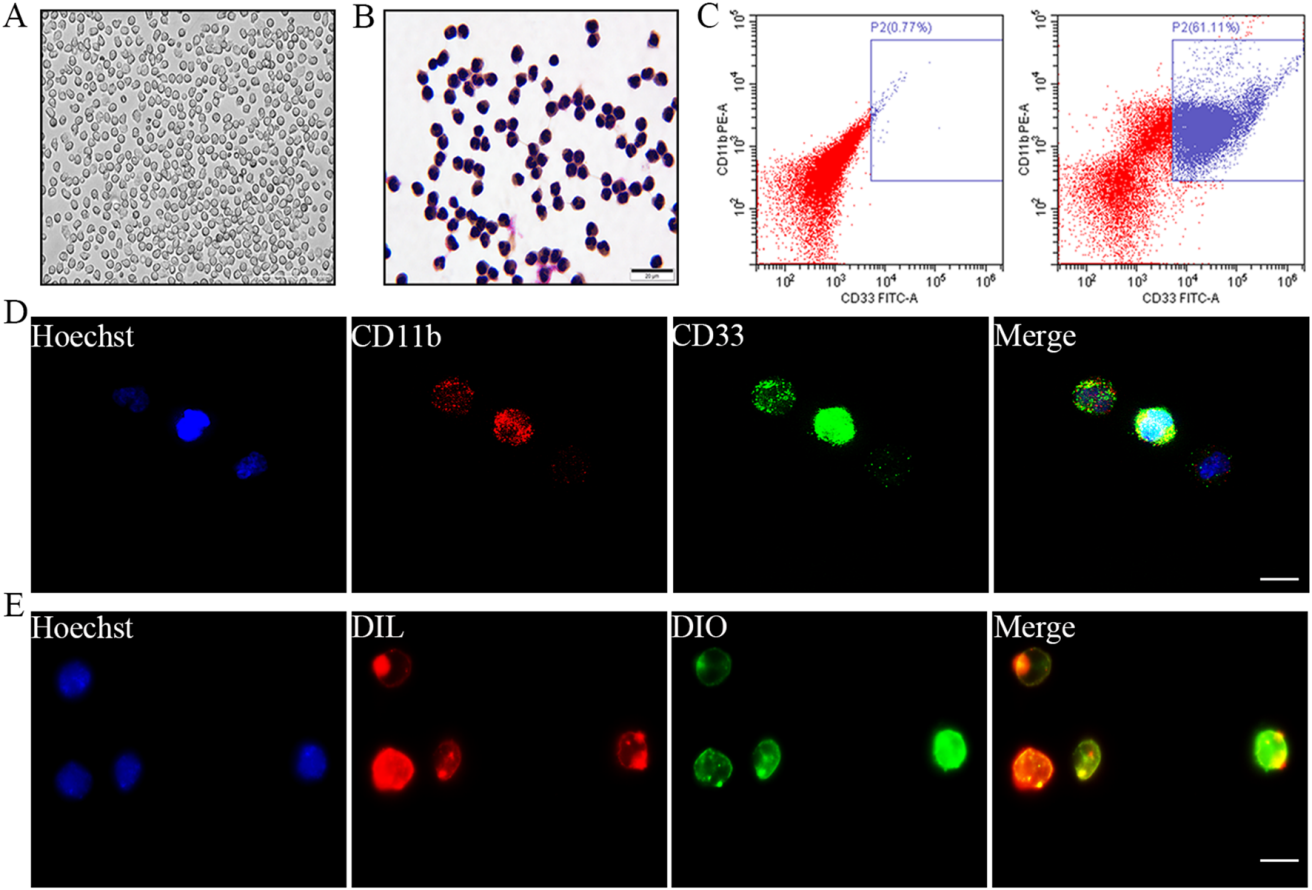
Isolation and characterization of neutrophils from human peripheral blood. **a** The morphological characteristics of neutrophils were detected by phase contrast microscopy (×200). **b** An orthographic microscope was used to detect the morphological characteristics of the neutrophils after Wright-Giemsa staining (×400). **c** Flow cytometry was performed to evaluate the surface characteristic antigens of the neutrophils. **d** Confocal microscopy was used to observe CD11b and CD33 antigens on the surface of the neutrophils (×600). **e** Laser scanning confocal microscopy was applied to identify the integrity of neutrophils labeled with DIO and DIL dyes (×600)

Peripheral blood neutrophils were isolated according to previous methods [24]. The isolated neutrophils had a uniform shape, spherical structure, and light-shielding (Fig. 2a). The neutrophils were deeply stained in the cytoplasm and had various nuclei, including rod and lobulated nuclei, and the neutrophil count results showed that the separation purity reached more than 95% (Fig. 2b). The flow cytometry and fluorescence staining showed that CD11b and CD33 were highly expressed on the surface of the neutrophil membrane, and neutrophils co-expressing CD11b and CD33 accounted for 61.11% of the total cells (Fig. 2c, d). In addition, the staining results of DIO and DIL showed that the neutrophils had a complete cell membrane structure (Fig. 2e).

The plasma membranes of the neutrophils were extracted as previously described with added modifications [25–27]. The neutrophils isolated by density gradient centrifugation were rapidly extracted for cell membrane proteins (Additional file 1: Figs. S3a and S4a). Coomassie blue staining showed that the fractions of neutrophil membrane proteins were distributed in the range of 15–250 kDa, and the main fraction was concentrated at 70 kDa (Additional file 1: Fig. S3b). The membrane protein fractions after sonication were sequentially extruded through polycarbonate membranes with different pore sizes to form neutrophil membrane-derived Neu-NVs. The TEM and AFM results showed that the Neu-NVs were relatively uniform in size and had a disc-like structure (Additional file 1: Fig. S3c and d). Moreover, the height of the Neu-NVs was mainly concentrated around 10 nm (Additional file 1: Fig. S3e). The Neu-NVs highly expressed protein molecules of the neutrophil membrane surface, such as CCL2, CXCR4, Fas, ICAM-1, integrin αV, and integrin β3 (Additional file 1: Fig. S3f). The particle size of the Neu-NVs was in the range of 30–200 nm, and the peak was concentrated at 146 nm. The membrane potential detection results showed that the Neu-NVs exhibited a negative zeta potential (Additional file 1: Fig. S3g, h). In addition, the fluorescence staining results also showed that DIO- and DIL-stained Neu-NVs had membrane vesicles (Additional file 1: Fig. S4b).

### Preparation and characterization of NEX

**Fig. 3:**
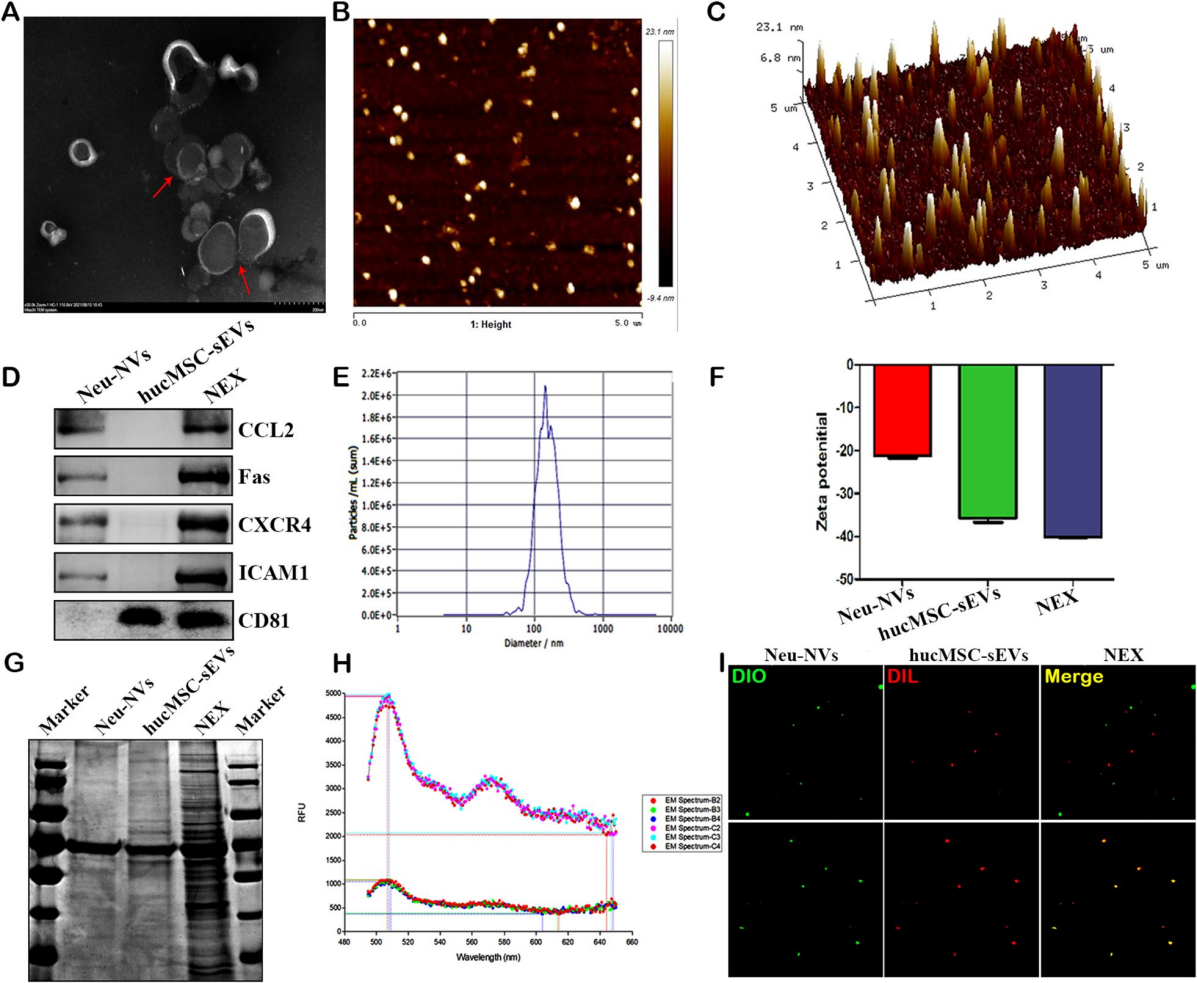
Preparation and characterization of NEX. **a** The size and morphology of NEX were detected by TEM. **b**, **c** The appearance and height of NEX were observed by AFM. **d** Characteristic protein molecules on the surface of NEX were measured by western blotting. **e**, **f** NTA was applied to verify the particle size, concentration, and zeta potential of NEX. **g** Protein components of NEX were detected by Coomassie blue staining. **h** Fluorescence resonance energy transfer experiments were performed to detect the fusion efficiency of NEX. **i** Super-resolution microscopy was used to detect the fusion efficiency of NEX (×600)

As one of the main inflammatory cells in the injury response, neutrophils highly express receptor ligands and chemokines, giving them natural inflammatory targeting. To solve the problem of insufficient targeting of hucMSC-sEVs, we used cell membrane fusion technology to obtain engineered hucMSC-sEVs. The results of TEM and AFM showed that hucMSC-sEVs could fuse with Neu-NVs to form NEX, which had a cup-shaped structure (Fig. 3a, b). Moreover, the height of NEX was concentrated around 10 nm (Fig. 3c). NEX co-expressed the characteristic proteins of neutrophils and hucMSC-sEVs (Fig. 3d). The particle size distribution of NEX was in the size range of 30–200 nm, and the peak was concentrated at 160 nm (Fig. 3e). In addition, NEX had a negative zeta potential, the absolute value of NEX membrane zeta potential is larger than that of Neu-NVs and hucMSC-sEVs alone, suggesting that.the fused NEX was more stable in solution (Fig. 3f). Coomassie blue staining showed that NEX protein components were distributed in the range of 15–250 kDa, the main component was 70 kDa, and both hucMSC-sEVs and Neu-NVs shared protein components (Fig. 3g). Fluorescence resonance energy transfer experiments were used to measure the fusion efficiency of the two vesicles. DIO dye and DIL dye have maximum emission signals at 501 nm and 565 nm, respectively (Additional file 1: Fig. S5a and b). When DIO-DIL-hucMSC-sEVs fused with Neu-NVs, the quenched DIO peak signal was remarkably recovered, as the physical distance between the two dyes increased (Fig. 3h and Additional file 1: Fig. S6). Moreover, compared with simple mixing, the DIO-labeled Neu-NVs and DIL-labeled hucMSC-sEVs were fused after sonication, and continuous efficient fusion occurred after extrusion; the fusion efficiency of NEX reached more than 95% (Fig. 3i and Additional file 1: Fig. S7). These results indicated that the hucMSC-sEVs had effective fusion with the Neu-NVs.

### NEX enhances the targeting of hucMSC-sEVs and effectively relieves cisplatin-induced acute renal damage

**Fig. 4:**
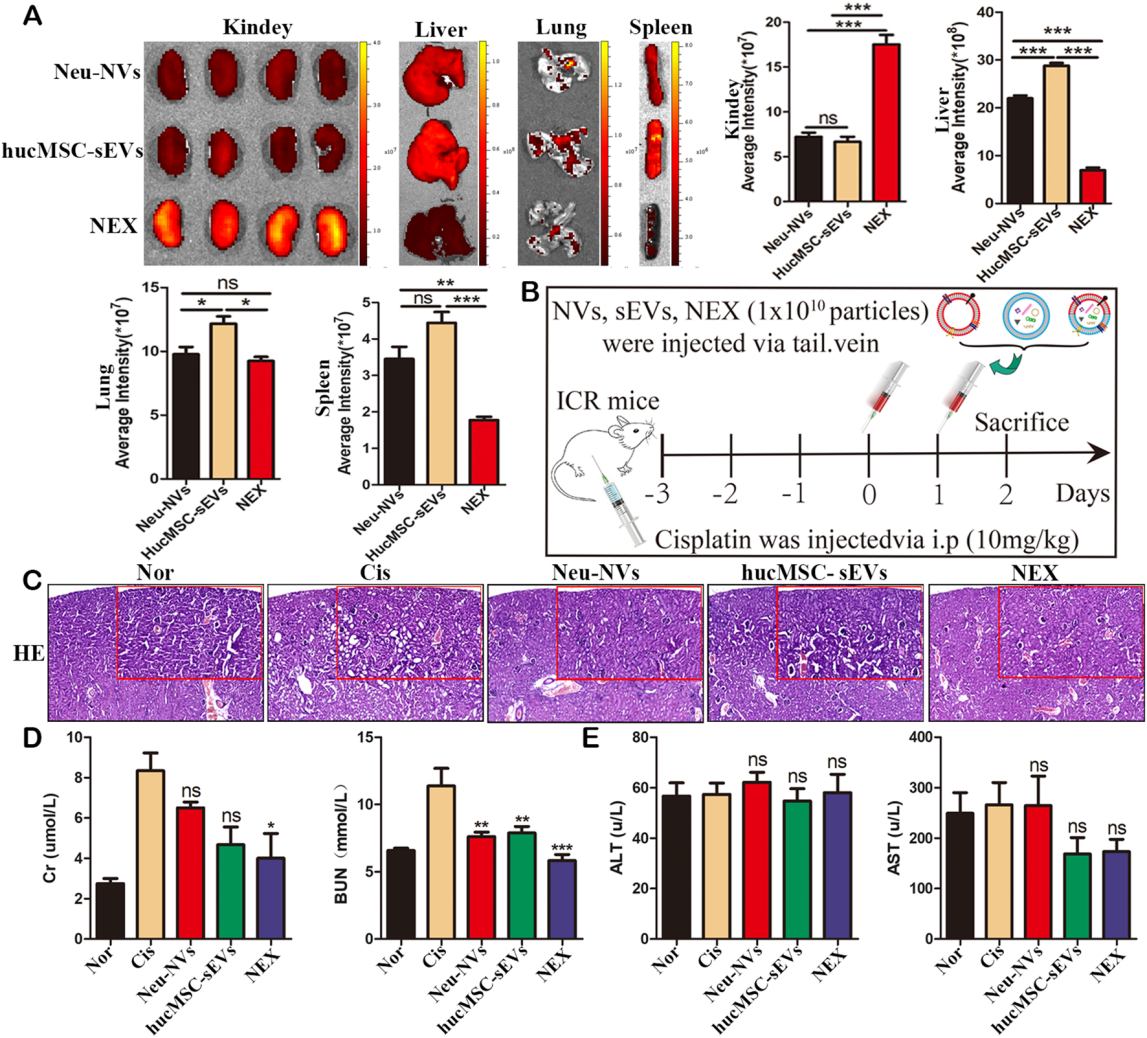
NEX enhances the targeting of hucMSC-sEVs and alleviates cisplatin-induced acute renal injury in vivo. **a** Small animal in vivo imaging was used to detect the distribution of three kinds of vesicles labeled with DIL dye in the cisplatin-induced acute kidney injury (AKI) mouse model. **b** Schematic diagram of three vesicles intervening in acute kidney injury induced by cisplatin. **c** HE staining was used to determine the pathological changes of renal tissues in the AKI model after three kinds of vesicle administration (scale bars: 100 μm and 200 μm). **d**, **e** Biochemical experiments were applied to evaluate the renal and liver function of the AKI model after three kinds of vesicle administration

Our previous studies verified that hucMSC-sEVs can effectively prevent cisplatin-induced acute kidney injury [13–16]. In this study, to investigate the targeting and therapeutic efficacy of neutrophil membrane-engineered hucMSC-sEVs, a cisplatin-induced AKI mouse model was established. The levels of renal function indexes Cr and BUN in the serum of the mice in the AKI model were significantly increased (Additional file 1: Fig. S8a), and pathological structural disorders were present in the kidney tissue, such as vacuolar degeneration, renal tubular epithelial cell necrosis, inflammatory cell infiltration, and cast formation (Additional file 1: Fig. S8b, c). To evaluate the targeting of NEX, the same number of DIL-labeled vesicles were administered to AKI model mice, and imaging was performed after 24 h of circulation in vivo. Compared with the Neu-NVs and hucMSC-sEVs groups, the NEX group had more obvious aggregation in the damaged kidney tissue, as well as significantly reduced aggregation in the liver and spleen enriched in the mononuclear phagocytosis system. In addition, the accumulation of NEX in the lungs was also significantly less than that of the Neu-NVs and hucMSC-sEVs groups (Fig. 4a). Subsequently, after 3 days of intraperitoneal injection of cisplatin, the mice were treated with Neu-NVs, hucMSC-sEVs, and NEX (Fig. 4b). Moreover, compared with the cisplatin-treated group, the renal histopathological structures of the mice in the Neu-NVs, hucMSC-sEVs, and NEX-treated groups were improved, and the NEX group had a more significant treatment effect (Fig. 4c). Compared with the model group, the Neu-NVs, hucMSC-sEVs, and NEX treatment groups significant decreased the renal function index BUN in serum, while only the NEX group decreased the expression level of Cr (Fig. 4d). Moreover, three kinds of vesicle treatment had no significant effect on the liver function indicators AST and ALT in the AKI model (Fig. 4e).

### NEX alleviates acute kidney injury via inhibiting the inflammatory response, promoting the proliferation of renal cells, and suppressing their apoptosis

**Fig. 5:**
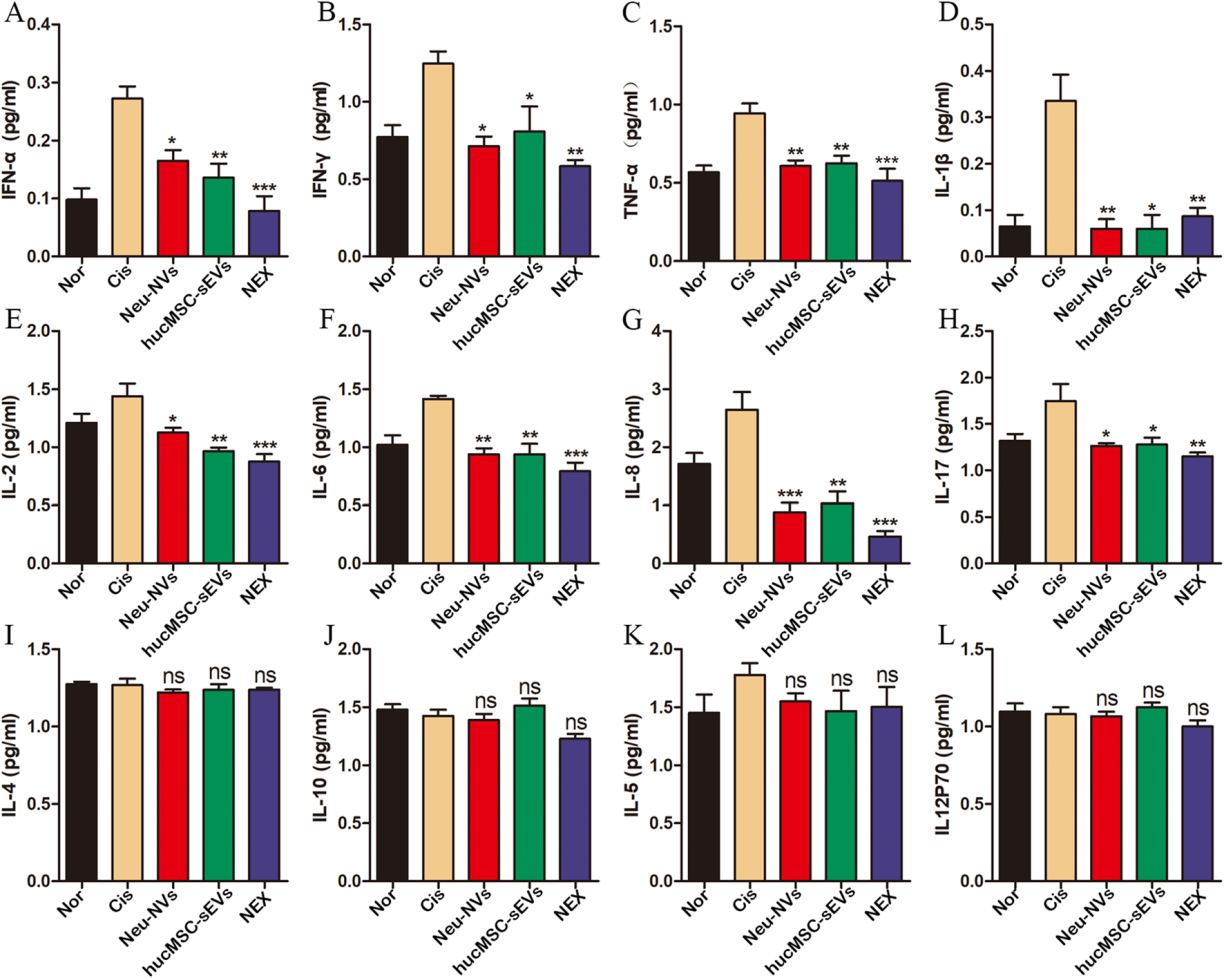
NEX decreases the level of serum inflammatory cytokines in the cisplatin-induced AKI mice model. **a-l** Multi-parameter flow cytometry was performed to detect the expression levels of serum proinflammatory cytokines, anti-inflammatory cytokines, and autoimmune-related factors in the AKI model after three kinds of vesicle administration. *p < 0.05, **p < 0.01, ***p < 0.001

**Fig. 6:**
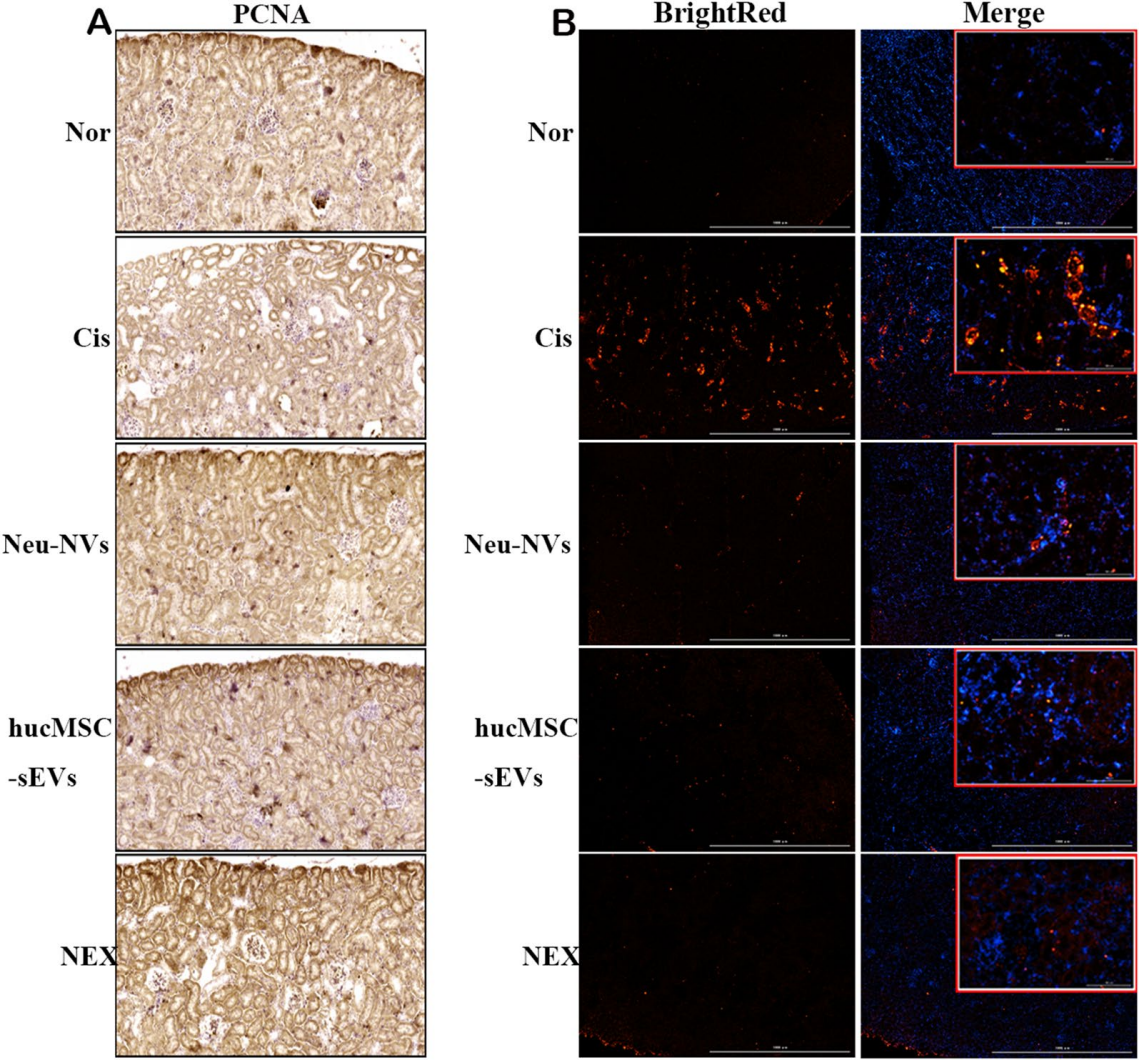
NEX alleviates cisplatin-induced acute renal injury and promotes the proliferation of renal tissue cells. **a** Immunohistochemical staining was used to evaluate the expression of PCNA in renal tissue cells in the AKI model after three kinds of vesicle administration (100 μm). **b** TUNEL staining was used to detect the apoptosis of renal tissue cells in in the AKI model after three kinds of vesicle administration (×40 and ×200)

To evaluate the therapeutic effect of NEX on AKI, multi-parameter flow cytometry was used to detect the expression of 12 cytokines in the serum of mice. Compared with the AKI model group, the Neu-NVs, hucMSC-sEVs, and NEX treatment groups effectively reduced the expression of pro-inflammatory cytokines including IFN-α, IFN-γ, TNF-α, IL-1β, IL-2, IL-6, IL-8, and IL-17 (Fig. 5a–h), while the expression of anti-inflammatory cytokines such as IL-4 and IL-10 (Fig. 5i, j) and autoimmune-related indicators such as IL-5 and IL-12P70 had not been significantly affected (Fig. 5h, l). These results suggest that Neu-NVs, hucMSC-sEVs, and NEX can effectively alleviate cisplatin-induced renal inflammation in mice, and the NEX treatment group had a more significant anti-inflammatory effect. In addition, the expression of PCNA was higher in the NEX treatment group, which suggested that NEX had a better effect on promoting the proliferation of renal tissue cells (Fig. 6b). The results of TUNEL staining showed that the Neu-NVs, hucMSC-sEVs, and NEX treatment groups all reduced the apoptosis of renal tissue cells in mice, while the NEX treatment group could better inhibit apoptosis (Fig. 6c).

### NEX promotes cellular uptake of NRK52E and their proliferation and alleviates cisplatin-induced cellular oxidative stress

**Fig. 7:**
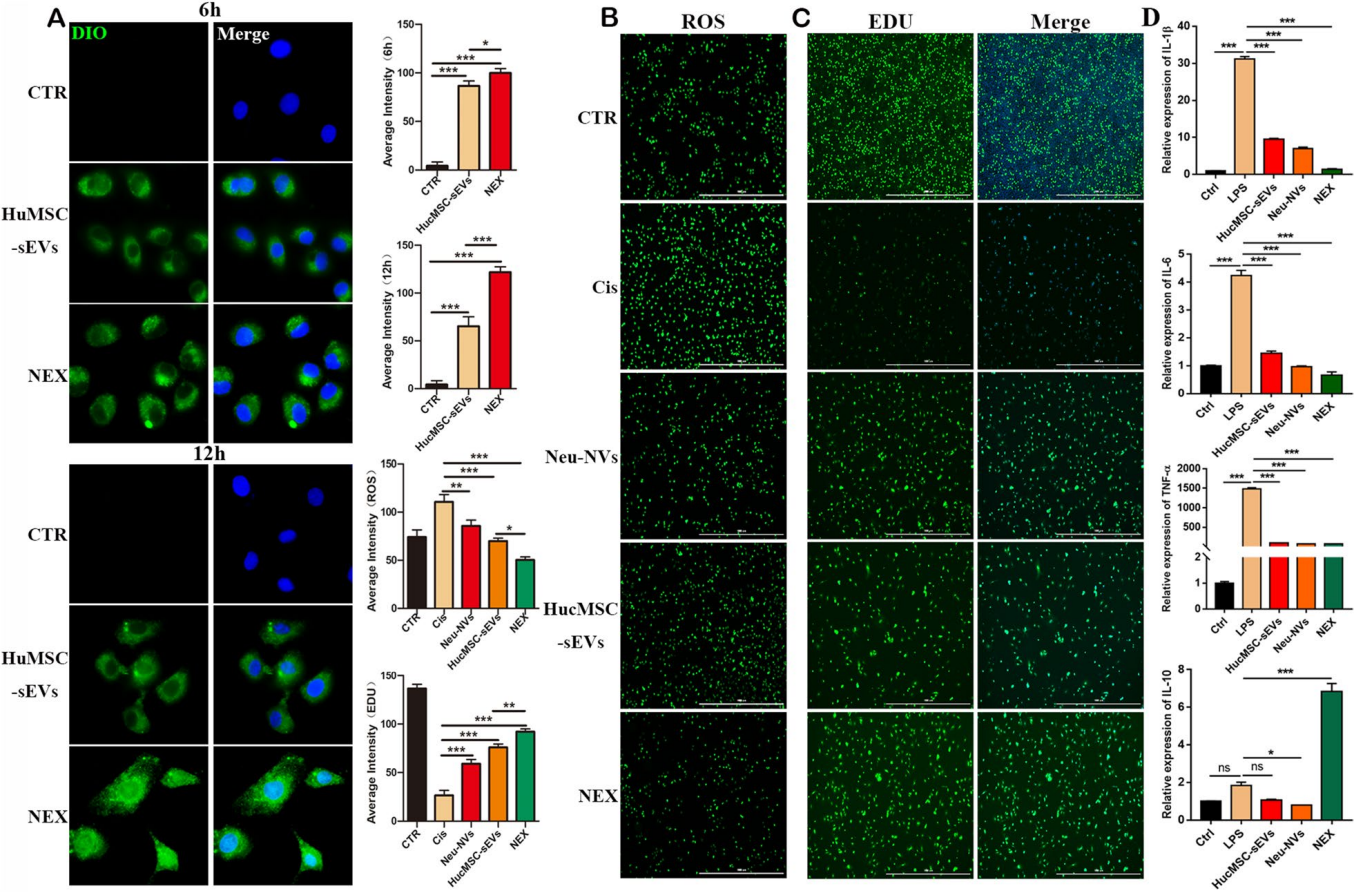
NEX promotes NRK52E cellular uptake and their proliferation, and decreases cisplatin-induced levels of oxidative stress. **a** Laser scanning confocal microscopy was used to detect the internalization of DIO-labeled hucMSC-sEVs and NEX by NRK52E cells at different time points (×600). **b** An automatic microplate reader was applied to determine the level of oxidative stress in NRK52E cells treated with cisplatin and three kinds of vesicles (×40) and **c** to evaluate the cell proliferation activity of NRK52E cells after cisplatin and three kinds of vesicle administration (×40). **d** qRT-PCR was used to detect the expression levels of proinflammatory and anti-inflammatory factors in NRK52E cells treated with three kinds of vesicles and LPS

To evaluate the targeting and therapeutic effect of NEX on renal tubular endothelial cells in vitro, we established a cisplatin-induced cell damage model. After treatment of NRK52E cells with different concentrations of cisplatin for 12 h, the cells underwent concentration-dependent apoptosis (Additional file 1: Fig. S9a). After 12 h and 24 h of cisplatin treatment, the cell proliferation activity was significantly inhibited in NRK52E cells (Additional file 1: Fig. S9b), and the expressions of pro-inflammatory factors IL-1β and IL-6 were significantly increased (Additional file 1: Fig. S9c). To evaluate the targeting of NEX to renal tubular endothelial cells, DIO-labeled hucMSC-sEVs and NEX were incubated with NRK52E cells for a period of time to detect their internalization. We found that compared with hucMSC-sEVs, DIO labeled Neu-NVs were more easily taken up by NRK52E cells at different time points (Additional file 1: Fig. S10). This suggests that protein molecules enriched in neutrophils may contribute to targeted uptake by cells. Furthermore, we also found that compared with hucMSC-sEVs, NRK52E cells could uptake NEX to a greater extent; especially, the internalization efficiency was more significant after co-incubation for 12 h (Fig. 7a). In addition, Neu-NVs, hucMSC-sEVs, and NEX treatment with the same number of particles all inhibited the level of cisplatin-induced cellular oxidative stress, while NEX could more significantly reduce the expression levels of ROS (Fig. 7b). Further research found that cisplatin significantly inhibited the proliferation of NRK52E cells, while Neu-NVs, hucMSC-sEVs, and NEX treatment could promote the proliferation of NRK52E cells, and the NEX treatment group had a stronger pro-proliferation effect (Fig. 7c). Futhermore, we found that LPS (lipopolysaccharide) treatment significantly increased NRK52E cell proinflammatory cytokines, including IL-1β, IL-6 and TNF-α, while all three vesicles inhibited the expression of these proinflammatory cytokines. In addition, compared with the other two treatment groups, the NEX treatment group not only significantly reduced the expression level of pro-inflammatory factor but also significantly promoted the expression level of anti-inflammatory factor IL-10 (Fig. 7d). These results indicated that the NEX treatment group had superior cell targeting and therapeutic efficacy than those of the hucMSC-sEVs treatment group.

### NEX inhibits its uptake by RAW264.7 macrophages

**Fig. 8:**
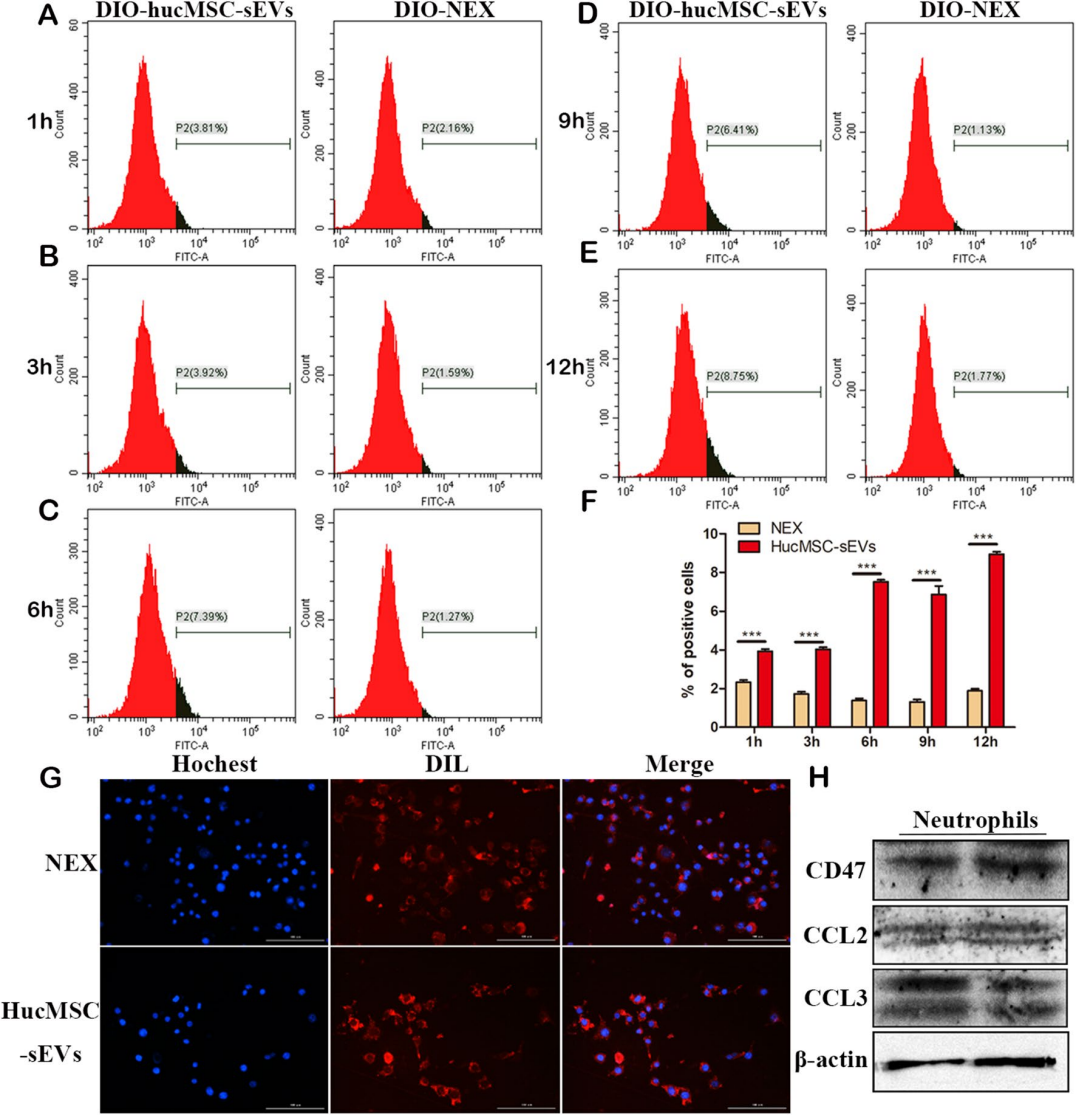
NEX inhibits uptake of hucMSC-sEVs by RAW264.7 macrophages. **a–e** RAW264.7 cellular uptake of DIO-labeled hucMSC-sEVs and NEX was detected by flow cytometry. **f** Statistical results of flow cytometry at different time points. **g** RAW264.7 cellular uptake of DIL-labeled hucMSC-sEVs and NEX was detected by the Cytation 5 automatic microplate reader. **h** Expression of the neutrophil CD47 molecule was detected using western blotting. *p < 0.05, **p < 0.01, ***p < 0.001

The underlying mechanisms of the reduction of NEX aggregation in the liver, spleen, and lung following infusion of Neu-NVs, hucMSC-sEVs, and NEX in vivo are unclear. We speculated that the neutrophil membrane may mediate the immune escape of hucMSC-sEVs in the mononuclear phagocytosis system. To test this hypothesis, DIO-labeled hucMSC-sEVs and NEX were co-incubated with RAW264.7 macrophages for different times. The flow cytometry results showed that DIO-labeled hucMSC-sEVs could be taken up by RAW264.7 macrophages in a time-dependent manner, but NEX inhibited this phenomenon (Fig. 8a–f). Moreover, after 24 h of DIL-labeled hucMSC-sEVs and NEX co-incubation with RAW264.7 macrophages, uptake of NEX by cells was significantly reduced (Fig. 8g). The results of western blotting showed that the neutrophils expressed large amounts of the molecule CD47, which send a “don’t eat me” signal to macrophages (Fig. 8h). However, CD47 was negatively expressed in hucMSC-sEVs (Additional file 1: Fig. S11). This result further suggests that neutrophil membrane protein components may be an important factor in mediating the immune escape of hucMSC-sEVs.

## Discussion

MSCs, as potential natural therapeutic cells, have been proven to treat a wide range of common and complex clinical diseases. MSC-based cell transplantation therapy has attracted extensive attention from researchers. HucMSCs are considered an ideal seed cell source in the field of tissue injury repair due to their advantages of abundant sources, easy access, wide application, and lack of ethical controversy. A large number of clinical and preclinical trials have proved that hucMSCs play an important role in the repair and treatment of tissue injury in vivo. However, the potential tumorigenic risk of long-term application of MSCs after transplantation in vivo and the difficulty of storage and transportation limit their wide clinical application. Therefore, there is an urgent need to find new biomaterials or stem-cell derivatives that can replace MSCs for tissue regeneration. As a derivative of MSCs, sEVs are key components of their paracrine. More studies have shown that hucMSC-sEVs have a significant therapeutic effect in various diseases. Currently, as a new alternative treatment strategy for “cell-free” disease, hucMSC-sEVs are attracting great interest in many biomedical fields. In addition, sEVs are considered to be a superior drug delivery vector due to their unique structure, composition, morphological characteristics, and superior physicochemical stability and biocompatibility. However, there are still many challenges to address before the clinical application of natural sEVs can be realized, such as insufficient sources, low yield and bioactivity, difficulty to obtain, low tissue targeting, and instability in blood circulation. Therefore, there is an urgent need to develop more effective methods to enhance the targeting and therapeutic efficacy of sEVs.

In order to improve the targeting and therapeutic efficacy of MSC-sEVs in tissue damage repair and anti-tumor applications, scientists are striving to establish a series of sEV engineering strategies, implementing the modification of sEV membranes and the loading of therapeutic active molecules [22]. Among them, the membrane fusion strategy is a novel method to engineer sEVs. The surface of a monocyte membrane has the characteristics of an inflammatory chemokine receptor. Ge et al. [30] used a membrane fusion strategy to prepare monocyte membrane-functionalized bone marrow MSC-sEVs (BMMSC-sEVs), which significantly improved the homing ability and therapeutic efficacy of the BMMSC-sEVs to damaged myocardium in a mouse model. In another study, researchers exploited the adhesion and migration-mediated properties of platelet membrane surface proteins and used a membrane fusion strategy to fuse platelet membranes with BMMSC-sEVs, which not only enhanced sEVs uptake by cardiomyocytes but also improved the targeting and therapeutic effect of sEVs on a myocardial infarction mouse model [31, 32]. Neutrophils express many kinds molecules on their membrane surface, such as ligands and chemotactic proteins. In the early stage of tissue injury, neutrophils are first recruited to the site of damaged tissue due to the release of high concentrations of chemokines at the injury site. To improve the targeting and therapeutic effect of hucMSC-sEVs on tissue injury repair, we used a membrane fusion technology, where chimeric NEX was prepared by the fusion of human blood Neu-NVs and hucMSC-sEVs.

**Fig. 9:**
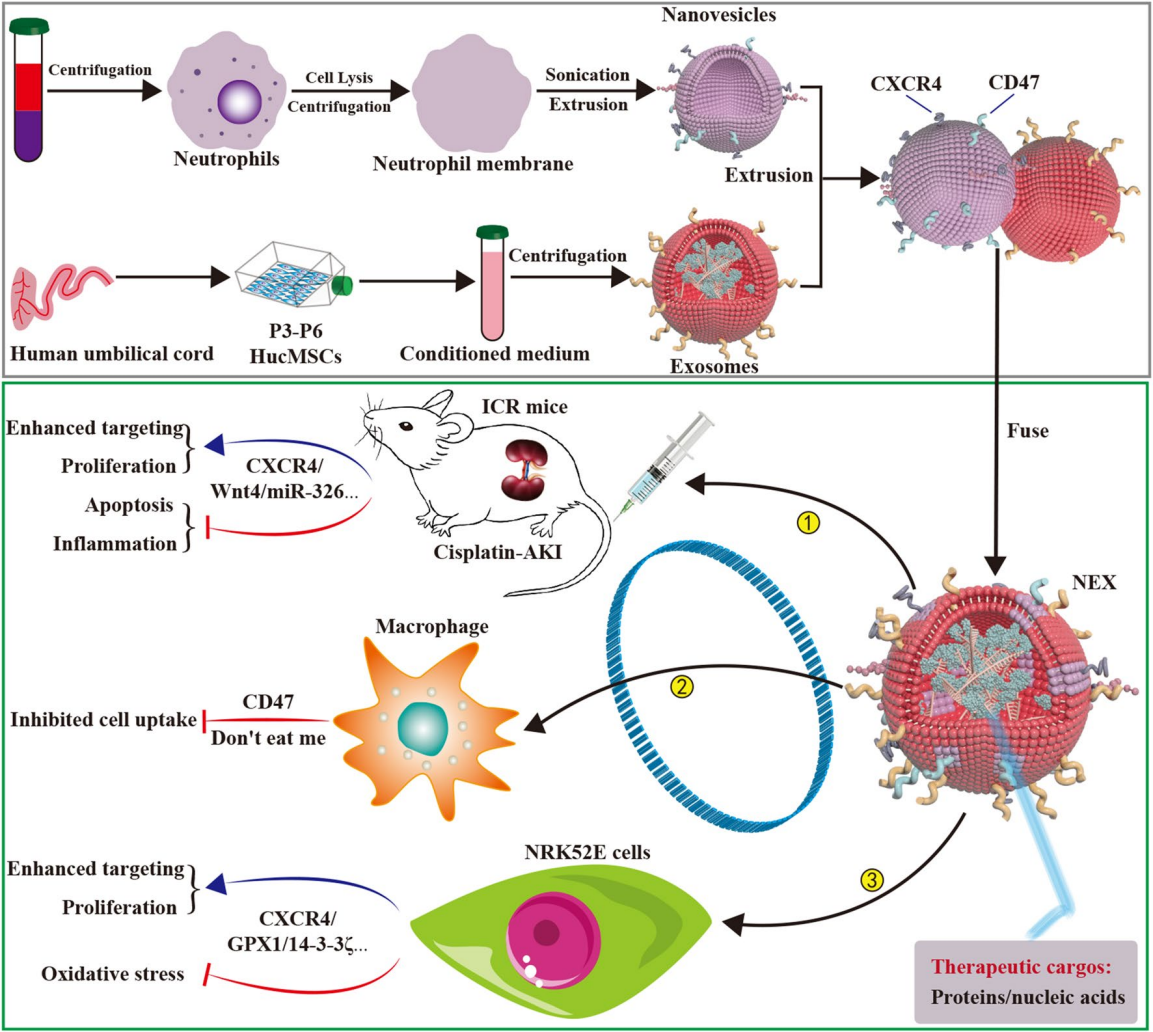
Schematic diagram of NEX preparation and its alleviating effect on cisplatin-induced acute kidney injury

Our previous results found that the 14-3-3ζ protein carried by huMSC-sEVs can be used to prevent and treat cisplatin-induced AKI, achieving positive effects. Our miRNA sequencing of hucMSC-sEVs showed that some of the proteins and miRNAs (including miR-125-5p, miR-146a-5p, miR-22-3p, miR-30c-5p, and miR-150-5p) known to repair AKI are enriched in hucMSC-sEVs (Additional file 1: Fig. S2b). To verify the targeting and the therapeutic efficacy of NEX in tissue damage repair, we established a cisplatin-induced AKI mouse model and cisplatin-induced NRK52E cell injury model for efficacy evaluation (Fig. 9). Three types of DIL-labeled vesicles were administered to AKI mice by tail vein injection. Small animals in vivo imaging found that NEX significantly enhanced the specific aggregation of hucMSC-sEVs in damaged kidney tissues, and it inhibited the retention of hucMSC-sEVs in liver and spleen tissues. Moreover, the three kinds of vesicles treatment groups all reduced the level of BUN, while only the NEX-treated group reduced Cr levels. Moreover, the three kinds of vesicles could inhibit various pro-inflammatory cytokines expression, while their had no effect on anti-inflammatory cytokines and autoimmunity-related cytokines expression, and compared with other treatment groups, the NEX group had a more significant anti-inflammatory effect. In addition, the three kinds of vesicle treatment groups all alleviated the renal tissue pathological changes caused by cisplatin, promoted the proliferation of renal tissue cells, and inhibited their apoptosis, and the therapeutic effect of NEX administration was more significant. These results indicate that NEX can efficiently target the site of renal tissue injury, effectively relieve cisplatin-induced renal function damage, promote renal tissue cell proliferation, and inhibit renal tissue cell inflammatory response and apoptosis in vivo.

To evaluate the targeting and therapeutic effect of NEX on target cells, a cisplatin-induced renal tubular endothelial cell injury model was established. Our findings showed that hucMSC-sEVs and NEX could be taken up by NRK52E cells in a time-dependent manner in vitro, the NEX exhibited a higher uptake efficiency. This suggests that the NEX enhanced the targeting of hucMSC-sEVs and promoted their uptake by renal tubular endothelial cells. Oxidative stress is an important factor in cisplatin-induced nephrotoxicity. To evaluate the therapeutic effect of NEX on the cisplatin-induced cellular oxidative stress injury model, three kinds of vesicles and cisplatin were co-treated in NRK52 cells. The results indicated that the three kinds of vesicles all promoted cell proliferation, with NEX exhibiting the strongest effect. Taken together, these results show that NEX had better cell targeting and therapeutic efficacy in vitro. However, the underlying mechanism by which NEX specifically aggregates in the injured kidney tissue to evade retention in the liver and spleen remains unclear. Studies have shown that the mononuclear phagocytosis system is an important reason for the decline in exosome utilization [27]. To explore whether NEX can mediate hucMSC-sEVs escaping from macrophage phagocytosis, DIO-labeled hucMSC-sEVs and NEX were incubated with RAW264.7 cells for different times. The results demonstrated that hucMSC-sEVs were internalized by RAW264.7 cells in a time-dependent manner, and NEX suppressed the uptake capacity of the macrophages. This suggests that NEX has a higher retention function to escape the monocyte-macrophage system, which is possibly related to neutrophil membrane protein components.

## Conclusion

NEX prepared by cell membrane fusion technology can improve the targeting and therapeutic efficiency of target cells, inhibit the phagocytosis of macrophages, and relieve cisplatin-induced acute renal function damage in vitro and in vivo. Our findings indicate that applying engineered hucMSC-sEVs is a new therapy for AKI, further suggesting that engineered hucMSC-sEVs have broader application prospects in tissue injury and repair. Finally, our results show that engineered exosomes also have broad application prospects.

## Supplementary Information

**Additional file 1: Table S1.** PCR primer sequences of genes. **Fig. S1.** Morphological characteristics, multipotential differentiation and surface marker identification of HucMSCs. **a** Inverted microscope was used to observe the morphological characteristics of primary and third generation hucMSCs (40×). **b** Oil red O staining and alizarin red S staining were performed to evaluate adipogenic differentiation (400×) and osteogenic differentiation of P3 hucMSCs (200×). **c** Flow cytometry were applied to detect the expression of hucMSCs surface specific antigens. **Fig. S2.** HucMSC-sEVs are highly enriched in various of beneficial molecules for AKI repair. **a** LC/MS–MS was performed to detect the proteomics of hucMSC-Ex. **b** Several molecules have been shown to be used for the repair of different acute kidney injury diseases. **Fig. S3.** Preparation and characterization of Neu-NVs. **a** Schematic diagram of preparation of human peripheral blood neutrophil membrane derived Neu-NVs. **b** Coomassie blue staining was performed to identify neutrophils and their cell membrane protein components. **c** The particle size and morphology of Neu-NVs were detected by TEM. **d, e** The appearance and height of Neu-NVs were observed by AFM. **f** Western blot was used to detect protein molecules on the surface of Neu-NVs. **g, h** NTA was applied to verify the particle size, concentration and Zeta potential of Neu-NVs. **Fig. S4.** Preparation and characterization of neutrophils membrane vesicles. **a** Neutrophils cell membrane precipitates were collected by differential supercentrifugation. **b** Immunofluorescence staining was perform to detect DIO and DIL-labeled Neu-NVs (600×). **Fig. S5.** DIO and DIL fluorescence emission spectra. **a,b** Automatic microplate reader cytation 5 was performed to detect DIO and DIL fluorescence emission spectra, respectively. **Fig. S6.** Fusion efficiency detection of Neu-NVs and hucMSC-sEVs. **a** The particle size and concentration of DIO-DIL-labeled hucMSC-sEVs and Neu-NVs were detected by NTA. **b** Fluorescence expression of DIO and DIL-labeled hucMSC-sEVs after fusion was detected by laser scanning confocal microscope (600×). **Fig. S7.** The particle size and concentration detection of DIO labeled Neu-NVs and DIL labeled hucMSC-sEVs. **a** The particle size and concentration of DIO labeled Neu-NVs were detected by NTA. **b** The particle size and concentration of DIL labeled hucMSC-sEVs were detected by NTA. **Fig. S8.** Construction of AKI model induced by cisplatin in vivo. **a** Biochemical assay was used to measure serum Cr and BUN of mice in normal group and AKI model group. **b** HE staining was performed to evaluate the pathological changes of renal tissues in normal group and AKI model group (100 μm). **c** Immunofluorescence staining as used to detect renal cell apoptosis in normal group and AKI model group (200×). **Fig. S9.** Construction of NRK52E cell injury model induced by cisplatin in vitro. **a** Cell apoptosis was detected by flow cytometry after NRK52E cells were treated with different concentrations of cisplatin. **b** CCK8 assay was applied to measure the proliferation activity of NRK52E cells treated with different concentrations of cisplatin for 12 h and 24 h. **c** qRT-PCR was used to detect the expression of proinflammatory cytokines IL-1β and IL-6 in NRK52E cells treated with 10 ng/mL cisplatin for 12 h. **Fig. S10.** DIO-labeled hucMSC-sEVs and DIO-labeled Neu-NVs were internalized by NRK52E cells. **a-c** Confocal microscopy was used to detect the cell internalization of DIO-labeled hucMSC-sEVs and DIO-labeled Neu-NVs after co-incubation with NRK52E cells for 6 h, 12 h and 24 h. **Fig. S11.** Detection of CD47 protein expression in sEVs derived from HucMSCs. Western blotting was used to detect characteristic protein markers and CD47 protein expression levels in HucMSC-sEVs.

## Data Availability

The data that support the conclusions of this study are available from the corresponding author upon request.

## Abbreviations

MSCs: Mesenchymal stem cells
HucMSCs: Human umbilical cord mesenchymal stem cells
ISEV: International Society for Extracellular Vesicles
EVs: Extracellular vesicles
sEVs: Small extracellular vesicles
MVs: Microvesicles
MVBs: Multivesicular bodies
HucMSC-sEVs: HucMSCs-derived sEVs
HFL1: Human embryonic lung fibroblasts
HFL1-sEVs: HFL1-derived sEVs
Neu: Neutrophil
Neu-NVs: Neutrophil membrane-derived Nanovesicles
NEX: Neu-NVs and hucMSC-Ex chimeric nanovesicles
IL-1β: Interleukin-1β
PCNA: Proliferating cell nuclear antigen
miRNA: MicroRNA
qRT-PCR: Quantitative real-time polymerase chain reaction
ROS: Reactive oxygen species
FBS: Fetal bovine serum
Cr: Creatinine
BUN: Urea nitrogen
