# Supplementary material for: Neutrophil membrane engineered HucMSC sEVs alleviate cisplatin-induced AKI by enhancing cellular uptake and targeting
Source: J Nanobiotechnology. 2022 Aug 1;20:353. doi: 10.1186/s12951-022-01574-8 (PMC9344666; doi:10.1186/s12951-022-01574-8)
Supplement: Supplementary file 1 — Additional file 1: Table S1. PCR primer sequences of genes. Fig. S1. Morphological characteristics, multipotential differentiation and surface marker identification of HucMSCs. a Inverted microscope was used to observe the morphological characteristics of primary and third generation hucMSCs (40×). b Oil red O staining and alizarin red S staining were performed to evaluate adipogenic differentiation (400×) and osteogenic differentiation of P3 hucMSCs (200×). c Flow cytometry were applied to detect the expression of hucMSCs surface specific antigens. Fig. S2. HucMSC-sEVs are highly enriched in various of beneficial molecules for AKI repair. a LC/MS–MS was performed to detect the proteomics of hucMSC-Ex. b Several molecules have been shown to be used for the repair of different acute kidney injury diseases. Fig. S3. Preparation and characterization of Neu-NVs. a Schematic diagram of preparation of human peripheral blood neutrophil membrane derived Neu-NVs. b Coomassie blue staining was performed to identify neutrophils and their cell membrane protein components. c The particle size and morphology of Neu-NVs were detected by TEM. d, e The appearance and height of Neu-NVs were observed by AFM. f Western blot was used to detect protein molecules on the surface of Neu-NVs. g, h NTA was applied to verify the particle size, concentration and Zeta potential of Neu-NVs. Fig. S4. Preparation and characterization of neutrophils membrane vesicles. a Neutrophils cell membrane precipitates were collected by differential supercentrifugation. b Immunofluorescence staining was perform to detect DIO and DIL-labeled Neu-NVs (600×). Fig. S5. DIO and DIL fluorescence emission spectra. a,b Automatic microplate reader cytation 5 was performed to detect DIO and DIL fluorescence emission spectra, respectively. Fig. S6. Fusion efficiency detection of Neu-NVs and hucMSC-sEVs. a The particle size and concentration of DIO-DIL-labeled hucMSC-sEVs and Neu-NVs were detected by NTA. b Fluorescence e [file 12951_2022_1574_MOESM1_ESM.docx]

**Neutrophil Membrane Engineered HucMSC sEVs Alleviate Cisplatin-induced AKI by Enhancing Cellular Uptake and Targeting**

Peipei Wu^1,2†^, Yuting Tang^1,2†^, Can Jin^1,2†^, Min Wang^1,2†^, Linli Li^1,2^, Zhong Liu^3^, Hui Shi^1,2^, Zixuan Sun^1,2^, Xiaomei Hou^1,2^, Wenya Chen^1,2^, Wenrong Xu^1,2^**^*^**, Hui Qian^1,2,4^**^*^**

^1^ Zhenjiang Key Laboratory of High Technology Research on Exosomes Foundation and Transformation Application, 301 Xuefu Road, Zhenjiang, Jiangsu, 212013, P.R. China.

^2^ Jiangsu Key Laboratory of Medical Science and Laboratory Medicine School of Medicine, Jiangsu University, 301 Xuefu Road, Zhenjiang, Jiangsu, 212013, P.R. China.

^3^ Department of Orthopedics, Shanghai General Hospital, School of Medicine, Shanghai Jiao Tong University, Shanghai 200080, China.

^4^ NHC Key Laboratory of Medical Embryogenesis and Developmental Molecular Biology & Shanghai Key Laboratory of Embryo and Reproduction Engineering, Shanghai 200040, China.

^†^ Peipei Wu, Yuting Tang, Can Jin and Min Wang contributed equally to this work.

***** Corresponding Authors

Wenrong Xu,Ph D

School of Medicine, Jiangsu University

301 Xuefu Road, 212013, Zhenjiang, Jiangsu, Republic of China

Telephone: +86 511 86102018, Fax: +86 511 86102010

E-mail: [icls@ujs.edu.cn](mailto:icls@ujs.edu.cn)

Hui Qian, Ph D

School of Medicine, Jiangsu University

301 Xuefu Road, Zhenjiang, Jiangsu, Republic of China

Telephone: +86 511 86102001, Fax: +86 511 86102010

E-mail: lstmmmlst@163.com

**Supplementary materials**

| Gene  name | Primer orientation | Sequence  （5’-3’） | product size | Annealing temperature |
| --- | --- | --- | --- | --- |
| β-actin | Forward | GACCTGTACGCCAACACAGT | 129 bp | 59 ℃ |
|  | Reverse | CTCAGGAGGAGCAATGATCT |  |  |
| Bax | Forward | CACCAGCTCTGAGCAGATCAT | 214 bp | 61 ℃ |
|  | Reverse | GATCAGTTCCGGCACCTTG |  |  |
| Bcl2 | Forward | GGATCCAGGATAACGGAGGC | 150 bp | 60 ℃ |
|  | Reverse | CCAGATAGGCACCCAGGGT |  |  |
| IL-1β | Forward | TTGAGTCTGCACAGTTCCCC | 160 bp | 60 ℃ |
|  | Reverse | TCCTGGGGAAGGCATTAGGA |  |  |
| IL-6 | Forward | AGAGACTTCCAGCCAGTTGC | 199 bp | 60 ℃ |
|  | Reverse | TGCCATTGCACAACTCTTTTC |  |  |

**Table. S1 PCR primer sequences of genes**

**Figures and Figure legends**

**
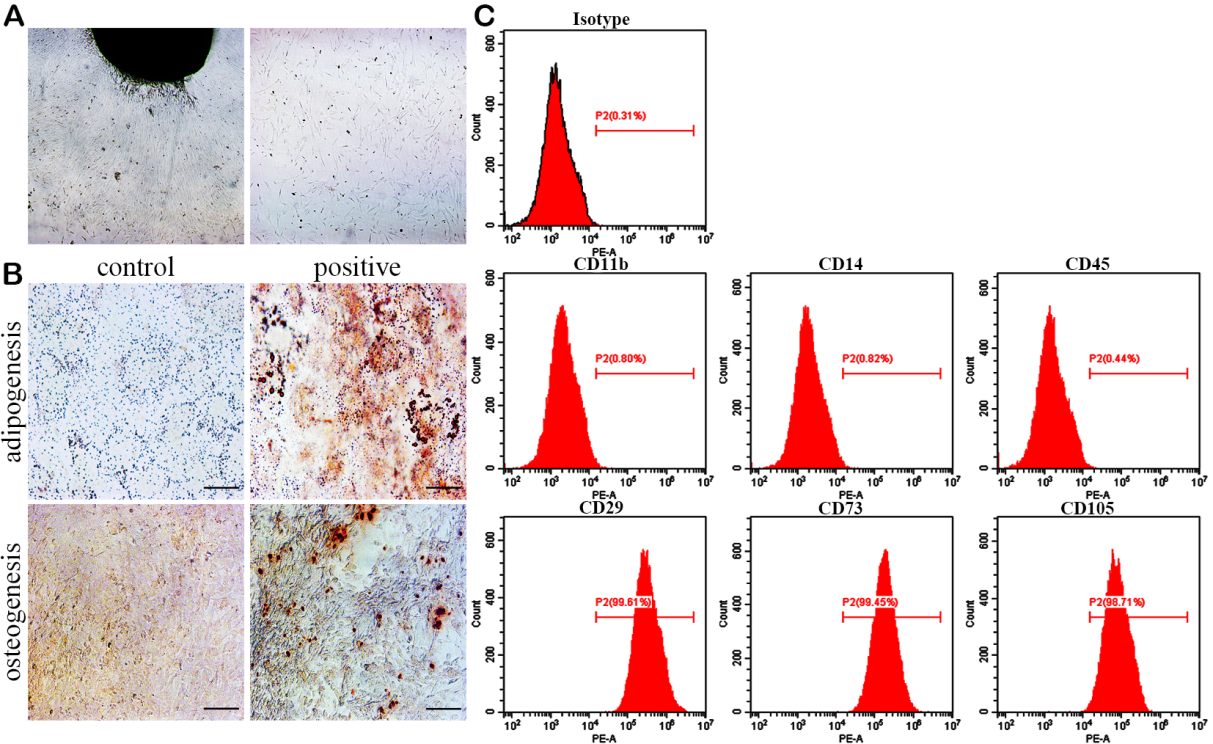
**

**Fig. S1** Morphological characteristics, multipotential differentiation and surface marker identification of HucMSCs. **a** Inverted microscope was used to observe the morphological characteristics of primary and third generation hucMSCs (40×). **b** Oil red O staining and alizarin red S staining were performed to evaluate adipogenic differentiation (400×) and osteogenic differentiation of P3 hucMSCs (200×). **c** Flow cytometry were applied to detect the expression of hucMSCs surface specific antigens.


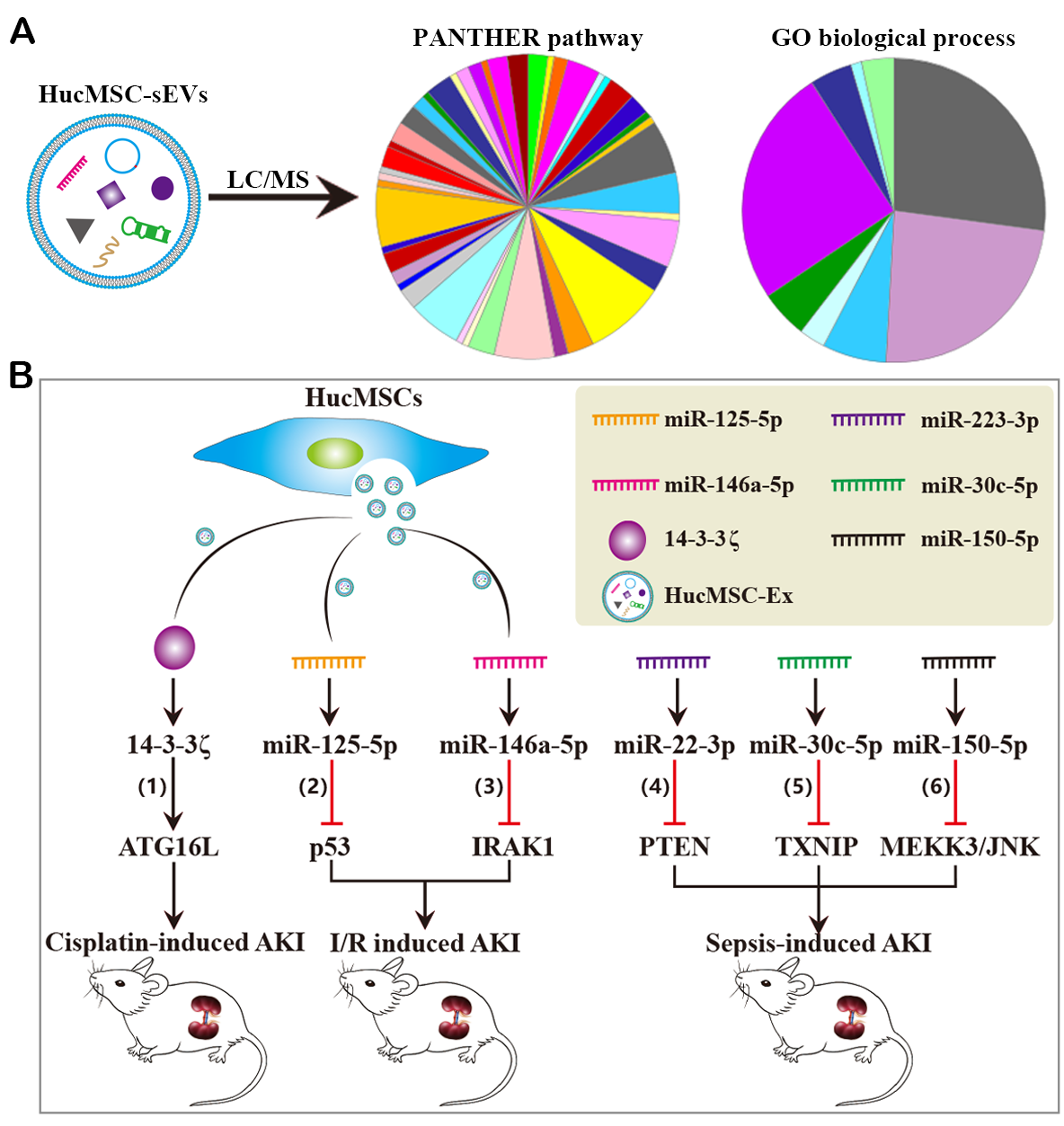


**Fig. S2** HucMSC-sEVs are highly enriched in various of beneficial molecules for AKI repair. **a** LC/MS-MS was performed to detect the proteomics of hucMSC-Ex. **b** Several molecules have been shown to be used for the repair of different acute kidney injury diseases.


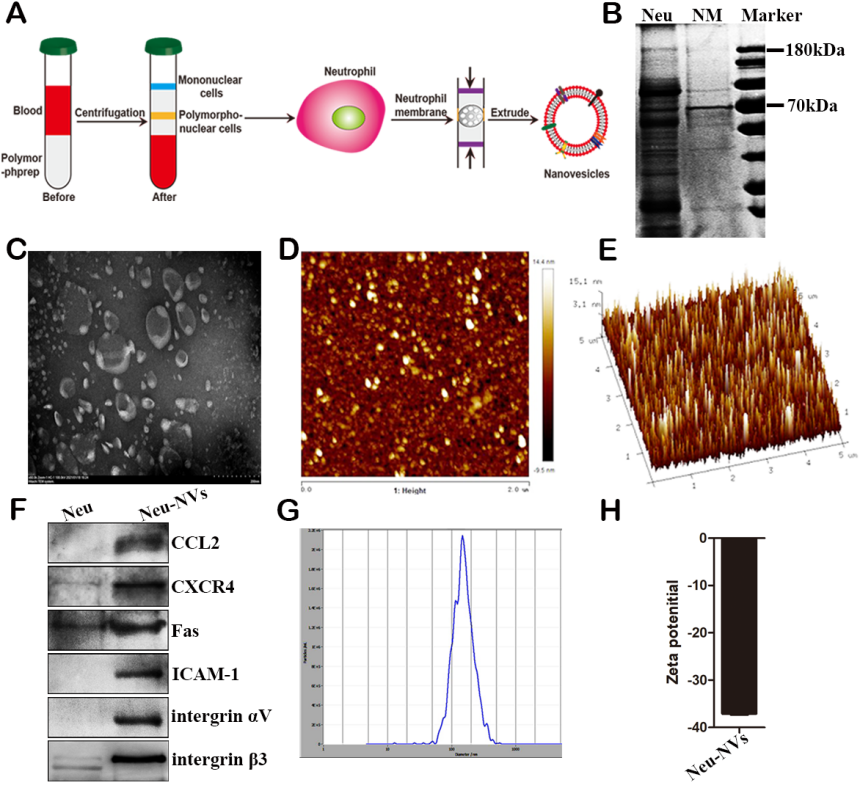


**Fig. S3** Preparation and characterization of Neu-NVs. **a** Schematic diagram of preparation of human peripheral blood neutrophil membrane derived Neu-NVs. **b** Coomassie blue staining was performed to identify neutrophils and their cell membrane protein components. **c** The particle size and morphology of Neu-NVs were detected by TEM. **d, e** The appearance and height of Neu-NVs were observed by AFM. **f** Western blot was used to detect protein molecules on the surface of Neu-NVs. **g, h** NTA was applied to verify the particle size, concentration and Zeta potential of Neu-NVs.


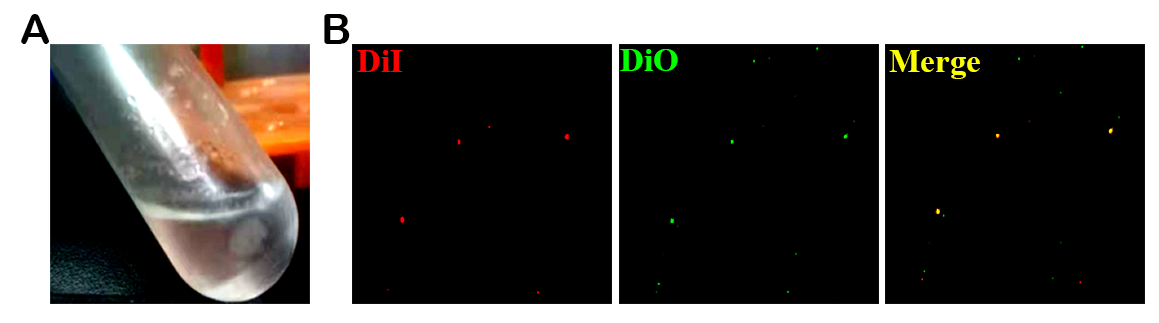


**Fig. S4** Preparation and characterization of neutrophils membrane vesicles. **a** Neutrophils cell membrane precipitates were collected by differential supercentrifugation. **b** Immunofluorescence staining was perform to detect DIO and DIL-labeled Neu-NVs (600×).


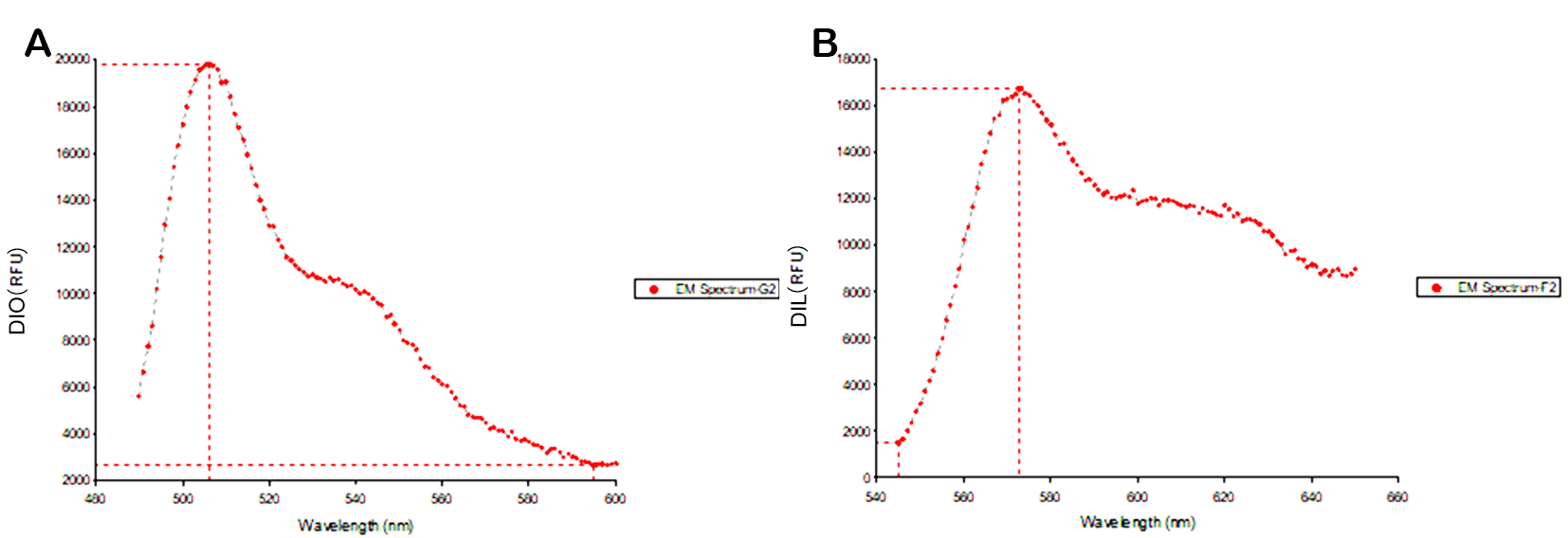


**Fig. S5** DIO and DIL fluorescence emission spectra. **a,b** Automatic microplate reader cytation 5 was performed to detect DIO and DIL fluorescence emission spectra, respectively.


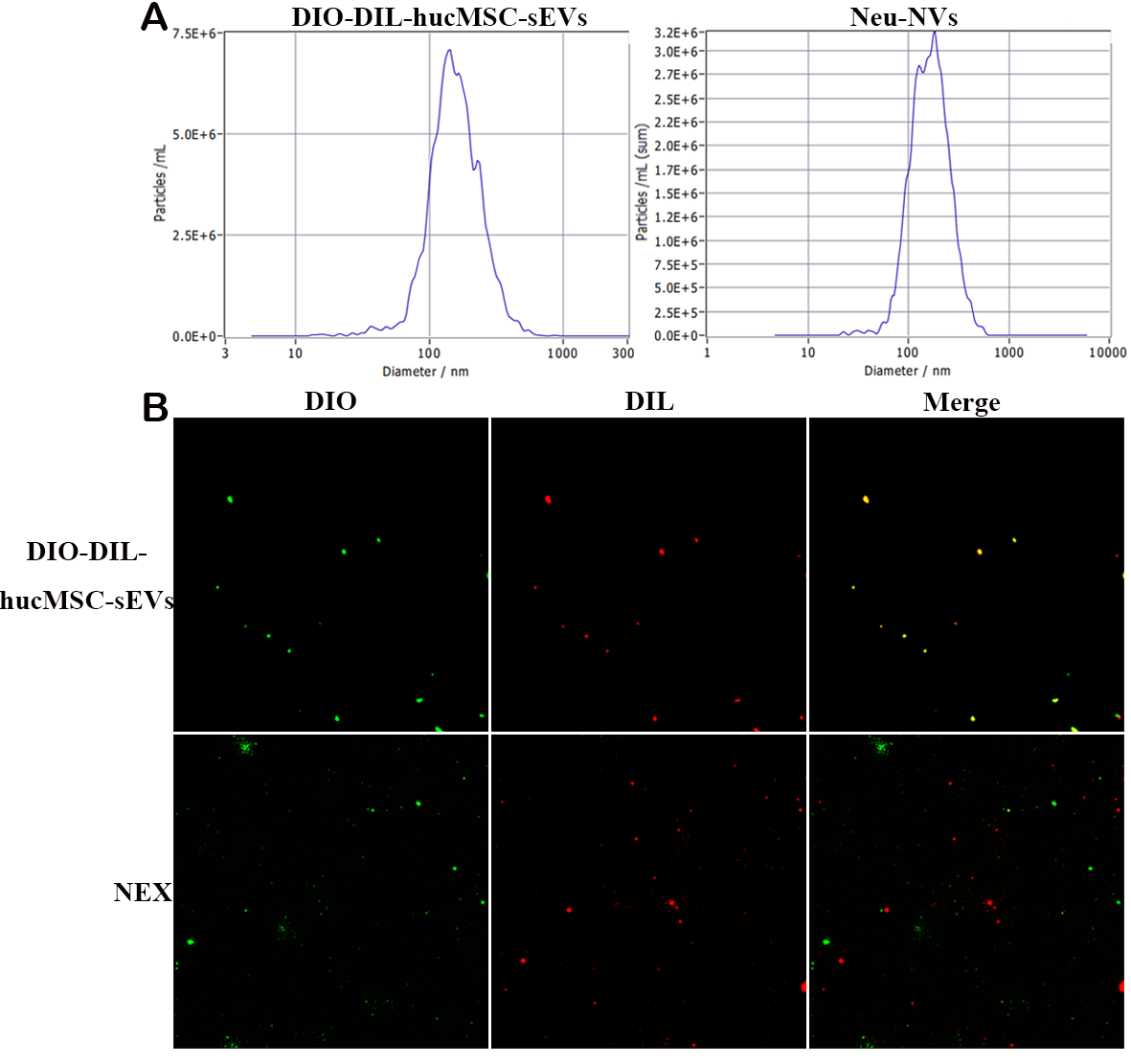


**Fig. S6** Fusion efficiency detection of Neu-NVs and hucMSC-sEVs. **a** The particle size and concentration of DIO-DIL-labeled hucMSC-sEVs and Neu-NVs were detected by NTA. **b** Fluorescence expression of DIO and DIL-labeled hucMSC-sEVs after fusion was detected by laser scanning confocal microscope (600×).


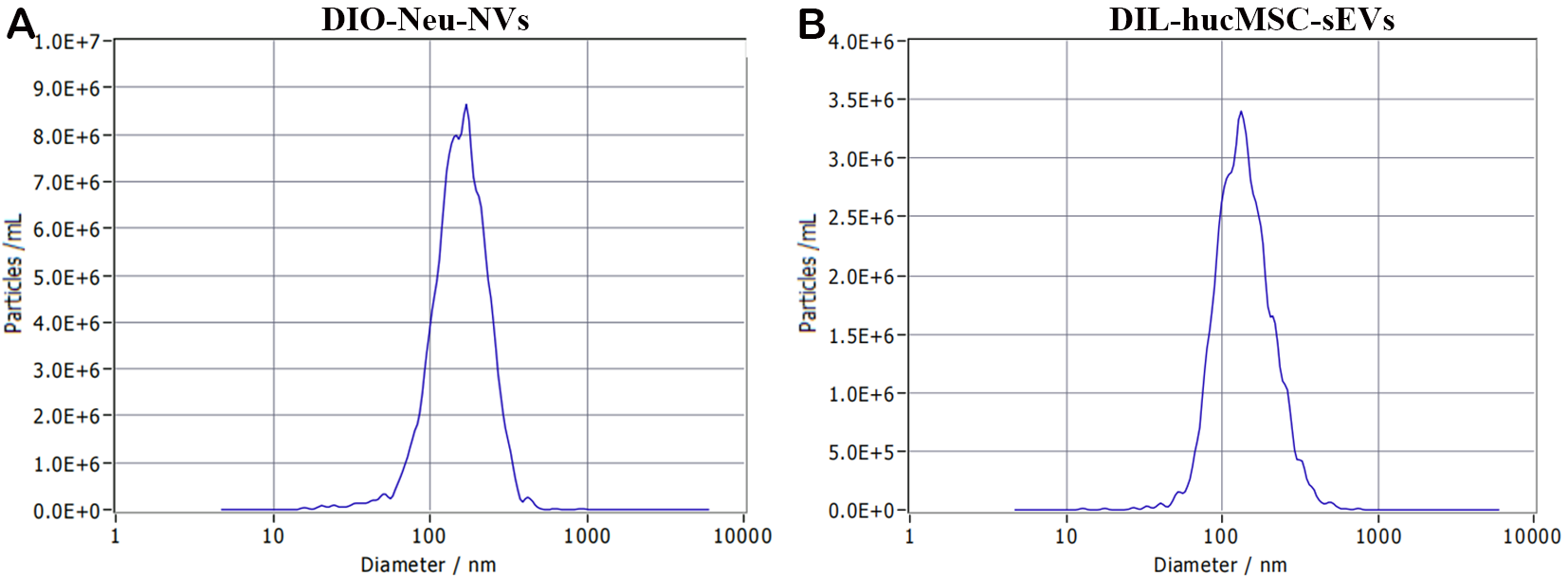


**Fig. S7** The particle size and concentration detection of DIO labeled Neu-NVs and DIL labeled hucMSC-sEVs. **a** The particle size and concentration of DIO labeled Neu-NVs were detected by NTA. **b** The particle size and concentration of DIL labeled hucMSC-sEVs were detected by NTA.


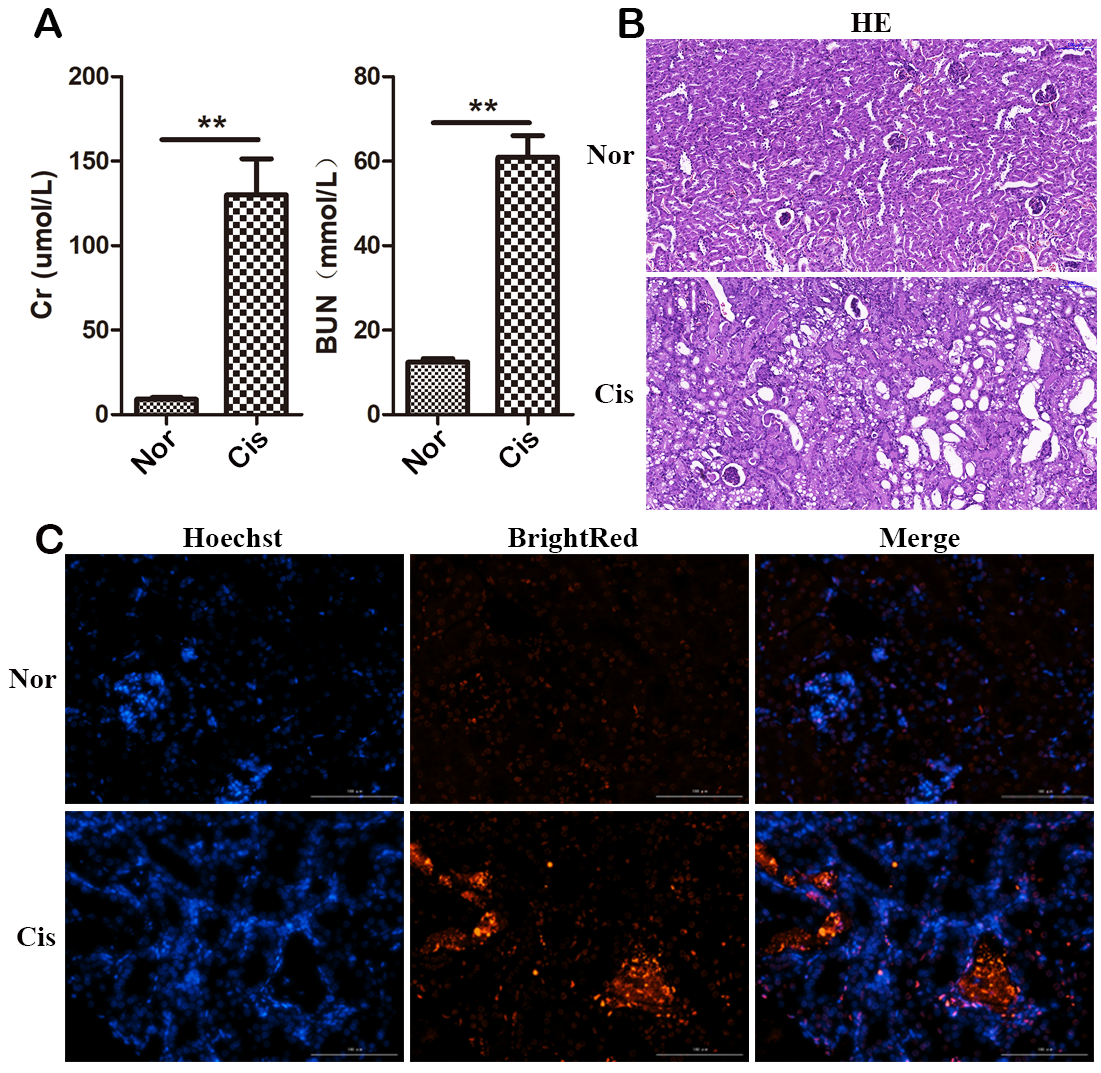


**Fig. S8** Construction of AKI model induced by cisplatin *in vivo*. **a** Biochemical assay was used to measure serum Cr and BUN of mice in normal group and AKI model group. **b** HE staining was performed to evaluate the pathological changes of renal tissues in normal group and AKI model group (100 μm). **c** Immunofluorescence staining was used to detect renal cell apoptosis in normal group and AKI model group (200×).


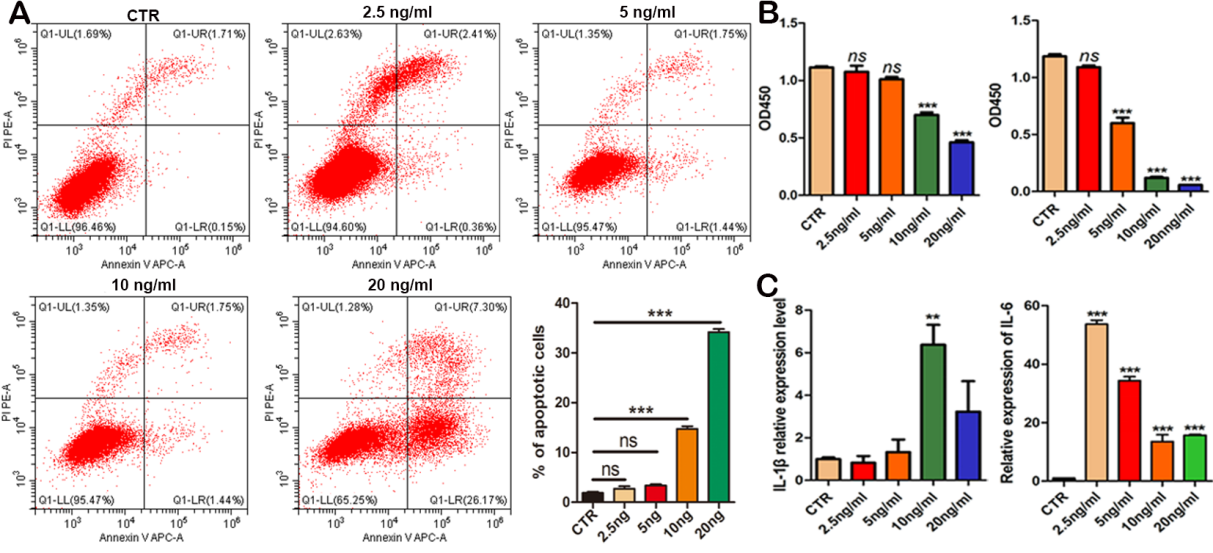


**Fig. S9** Construction of NRK52E cell injury model induced by cisplatin *in vitro*. **a** Cell apoptosis was detected by flow cytometry after NRK52E cells were treated with different concentrations of cisplatin. **b** CCK8 assay was applied to measure the proliferation activity of NRK52E cells treated with different concentrations of cisplatin for 12 h and 24 h. **c** qRT-PCR was used to detect the expression of proinflammatory cytokines IL-1β and IL-6 in NRK52E cells treated with 10 ng/mL cisplatin for 12 h.

**
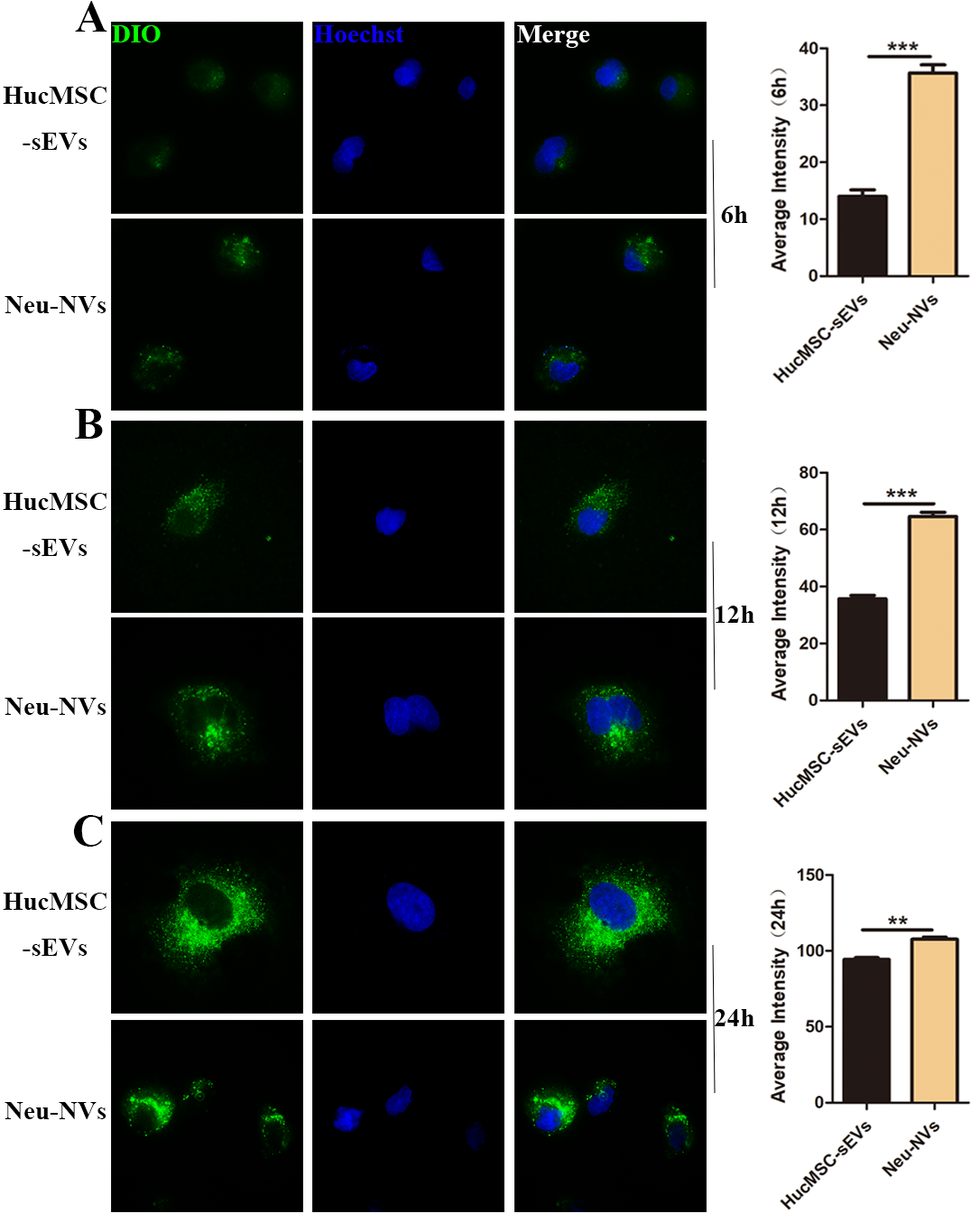
**

**Fig. S10** DIO-labeled hucMSC-sEVs and DIO-labeled Neu-NVs were internalized by NRK52E cells. **a-c** Confocal microscopy was used to detect the cell internalization of DIO-labeled hucMSC-sEVs and DIO-labeled Neu-NVs after co-incubation with NRK52E cells for 6h, 12h and 24h.


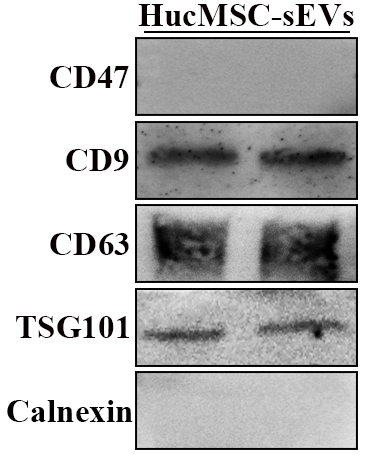


**Fig. S11** Detection of CD47 protein expression in sEVs derived from HucMSCs. Western blotting was used to detect characteristic protein markers and CD47 protein expression levels in HucMSC-sEVs.
